# Supplementary material for: Structural and quantum chemical analysis of exciton coupling in homo- and heteroaggregate stacks of merocyanines
Source: Nat Commun. 2016 Sep 29;7:12949. doi: 10.1038/ncomms12949 (PMC5056423; doi:10.1038/ncomms12949)
Supplement: Supplementary Information — Supplementary Figures 1-20, Supplementary Tables 1-9, Supplementary Notes 1-2, Supplementary Discussion, Supplementary Methods and Supplementary References [file ncomms12949-s1.pdf]

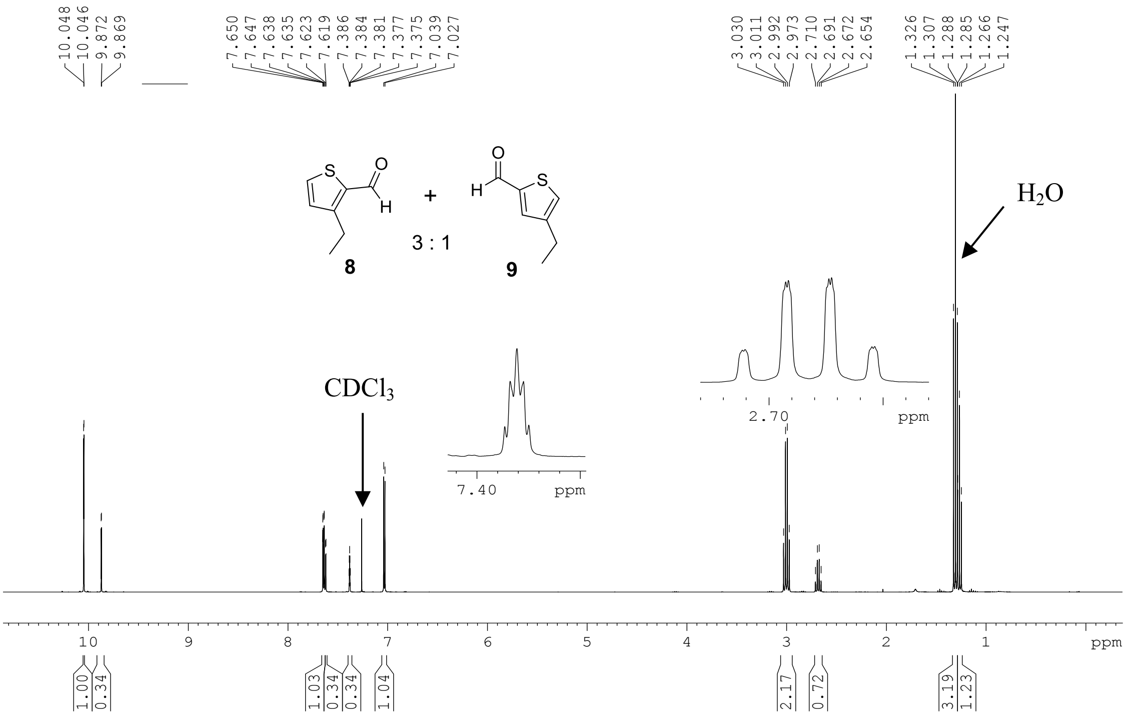

Supplementary Figure 1 | ^{1}H NMR (400 MHz) spectrum of a 3:1 mixture of 3-ethylthiophene-2-carboxaldehyde (8) and 4-ethylthiophene-2-carboxaldehyde (9) in $CDCl_3$ at 295 K.

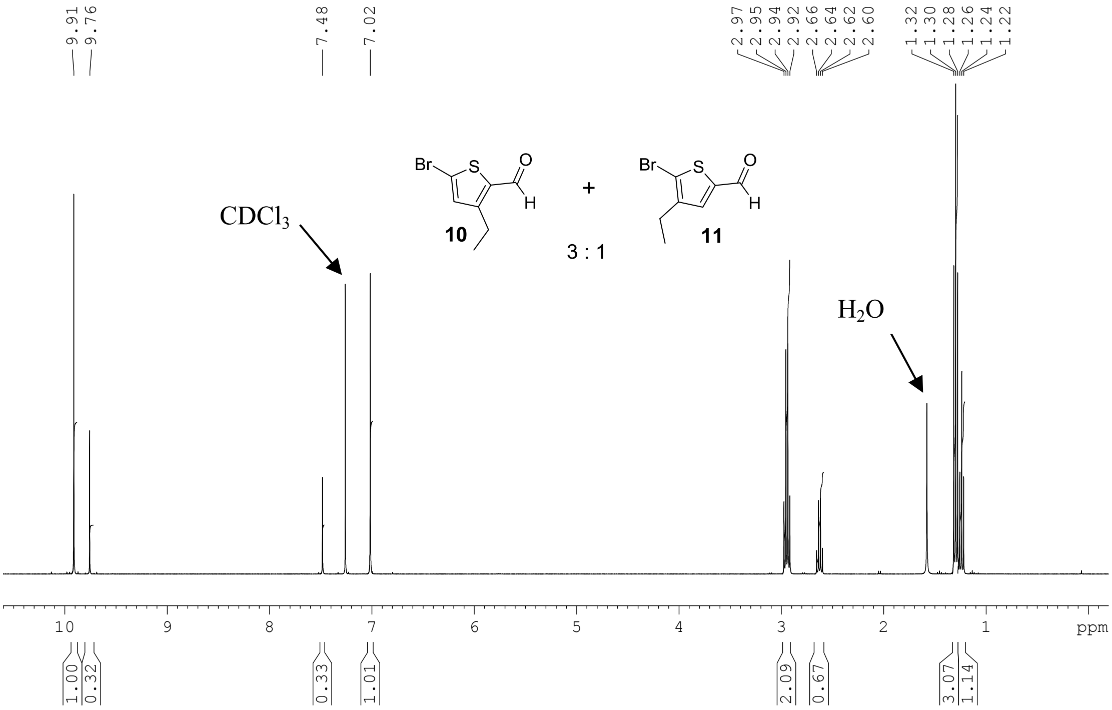

Supplementary Figure 2 | ^{1}H NMR (400 MHz) spectrum of a 3:1 mixture of 5-bromo-3-ethylthiophene-2-carboxaldehyde (10) and 5-bromo-4-ethylthiophene-2-carboxaldehyde (11) in $CDCl_3$ at 295 K.

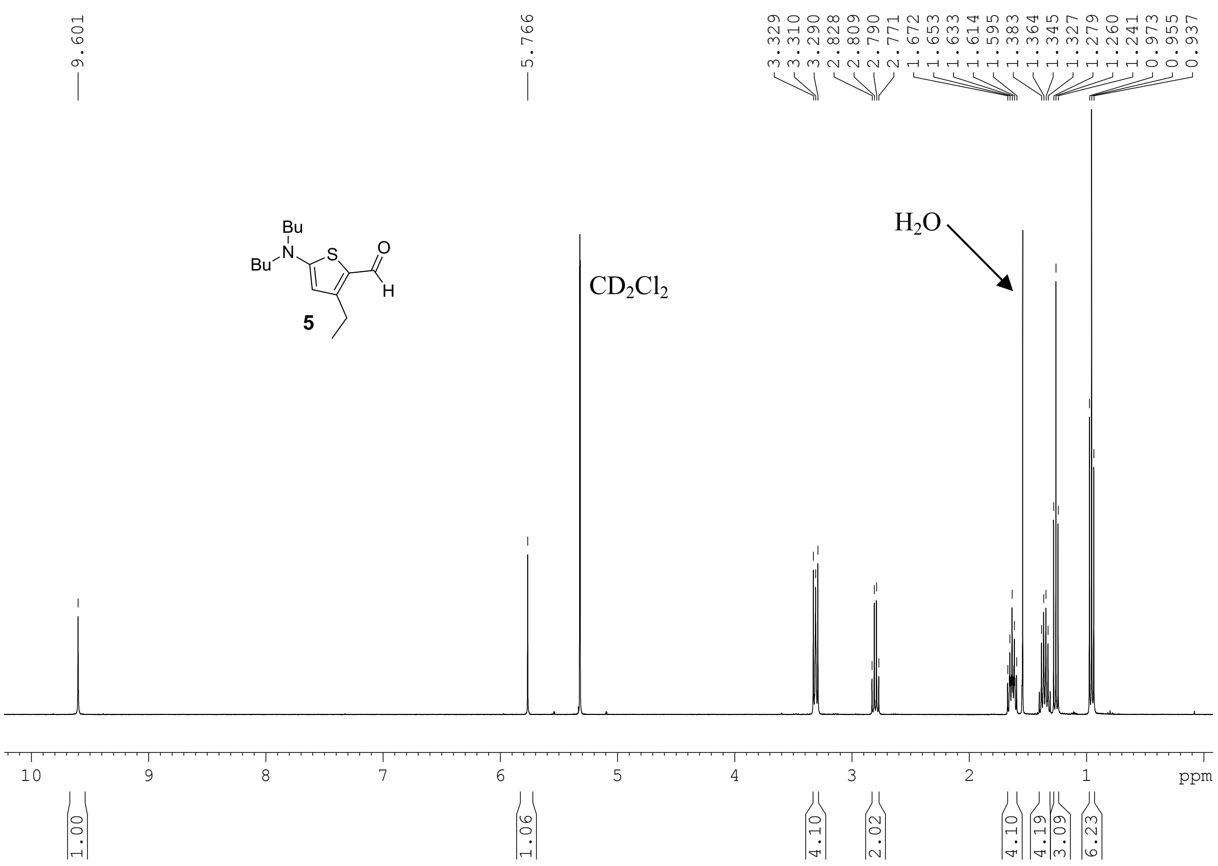

Supplementary Figure 3 | ^{1}H NMR (400 MHz) spectrum of 5 in CD_2Cl_2 at 295 K.

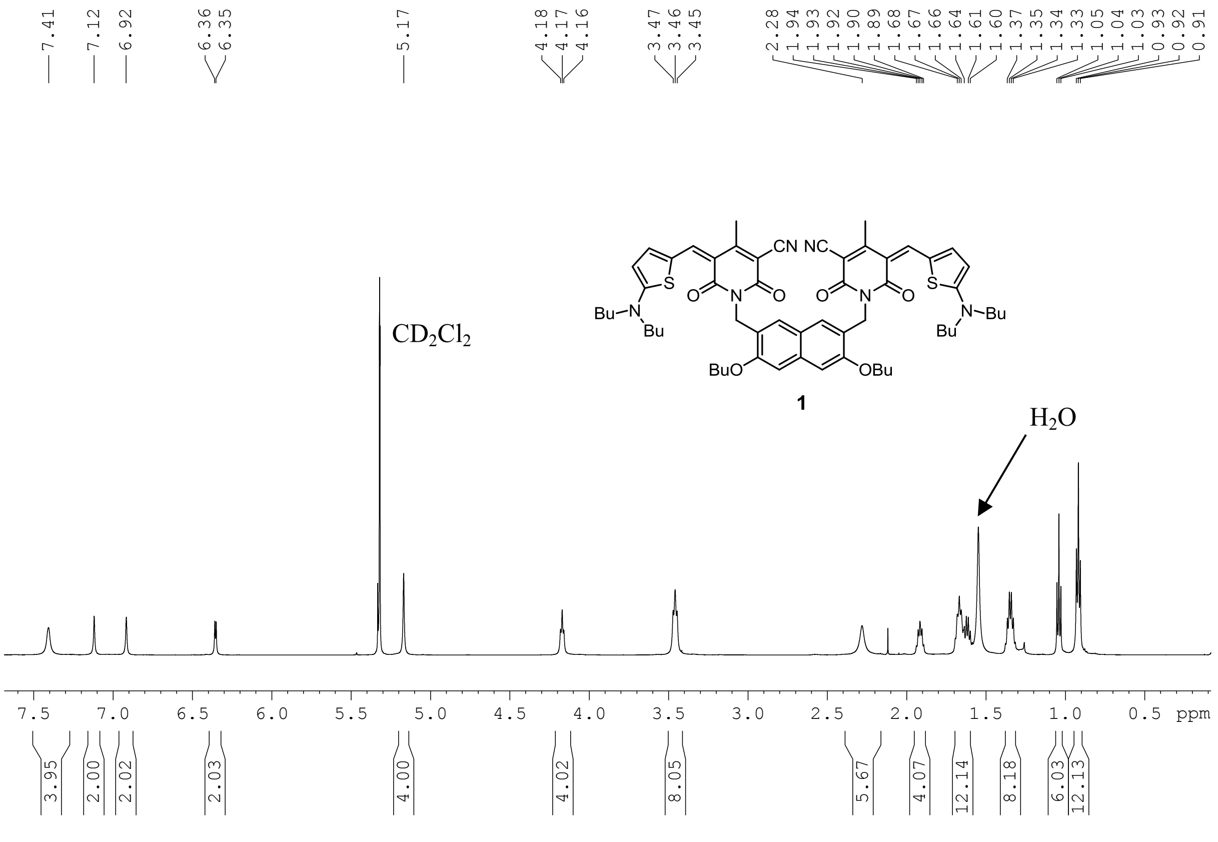

Supplementary Figure 4 | ^{1}H NMR (600 MHz) spectrum of bis(merocyanine) 1 in CD_2Cl_2 at 295 K.

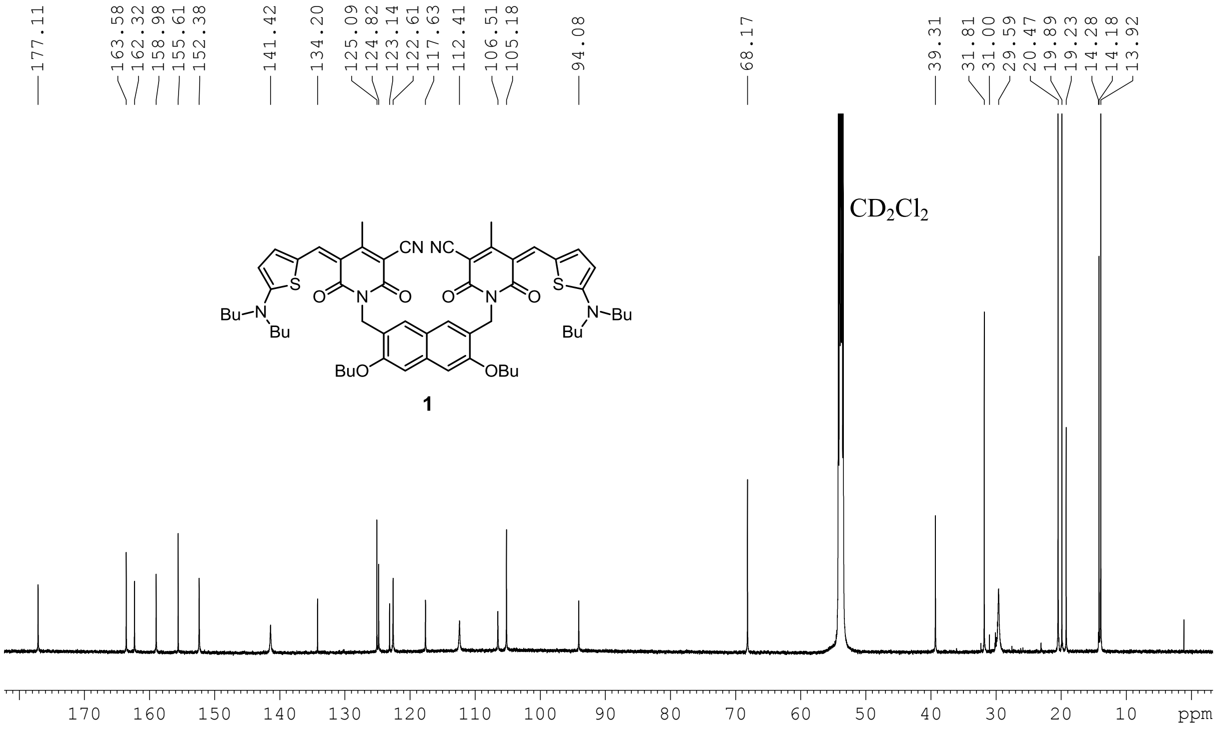

Supplementary Figure 5 | ^{13}C NMR (151 MHz) spectrum of 1 in CD_2Cl_2 at 295 K.

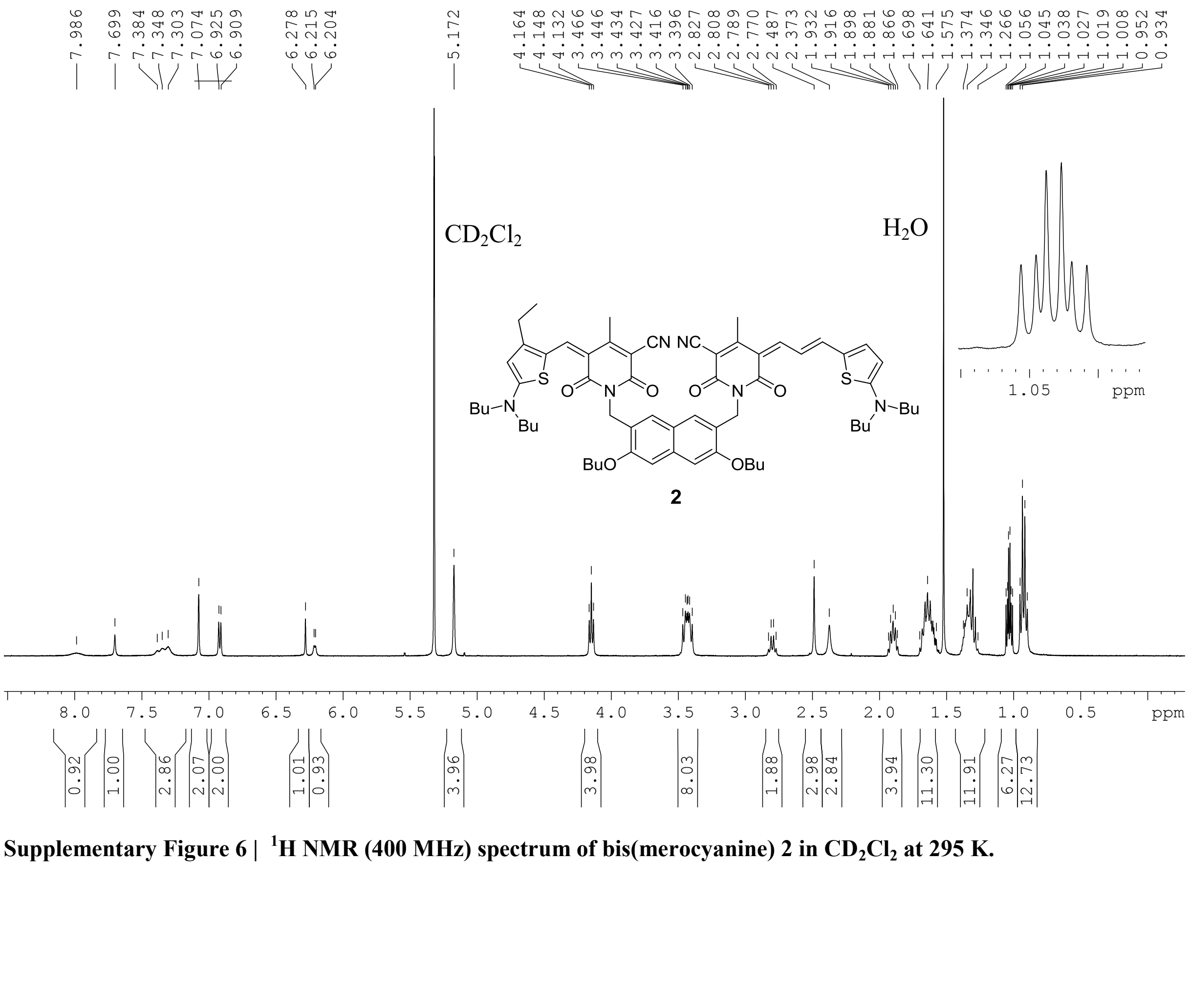

Supplementary Figure 6 | ^{1}H NMR (400 MHz) spectrum of bis(merocyanine) 2 in CD_2Cl_2 at 295 K.

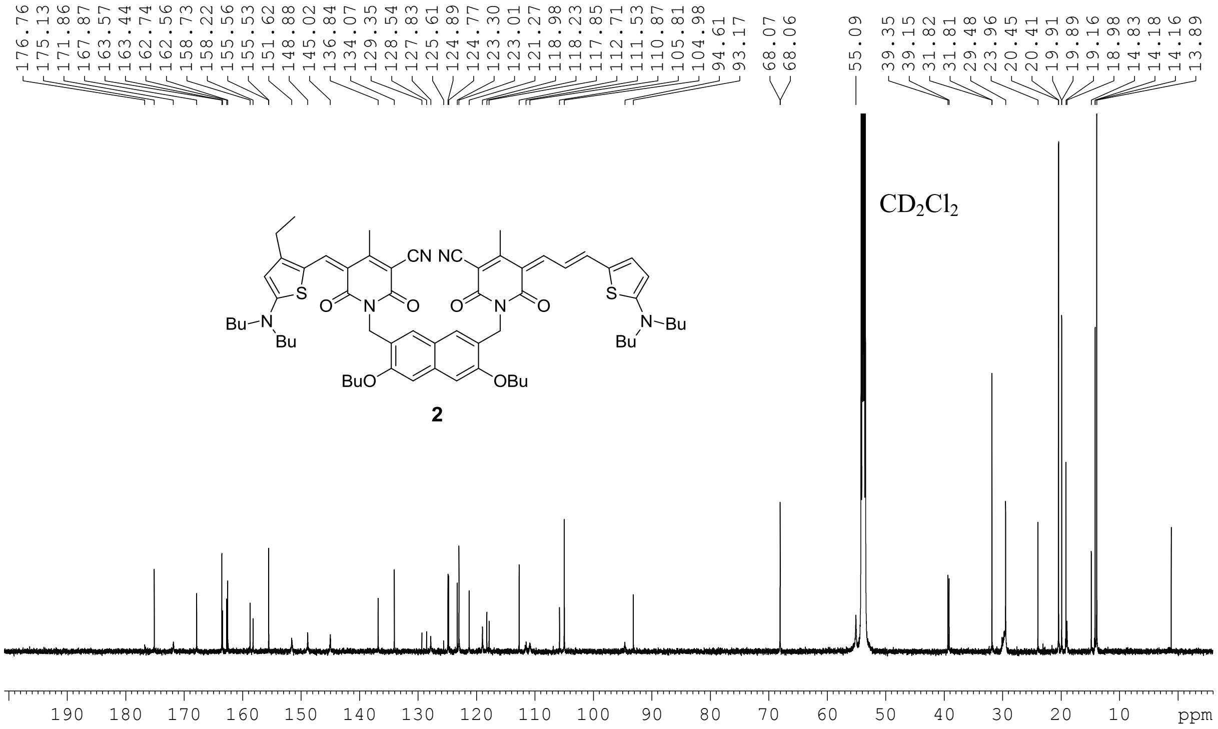

Supplementary Figure 7 | ^{13}C NMR (151 MHz) spectrum of bis(merocyanine) 2 in CD_2Cl_2 at 295 K.

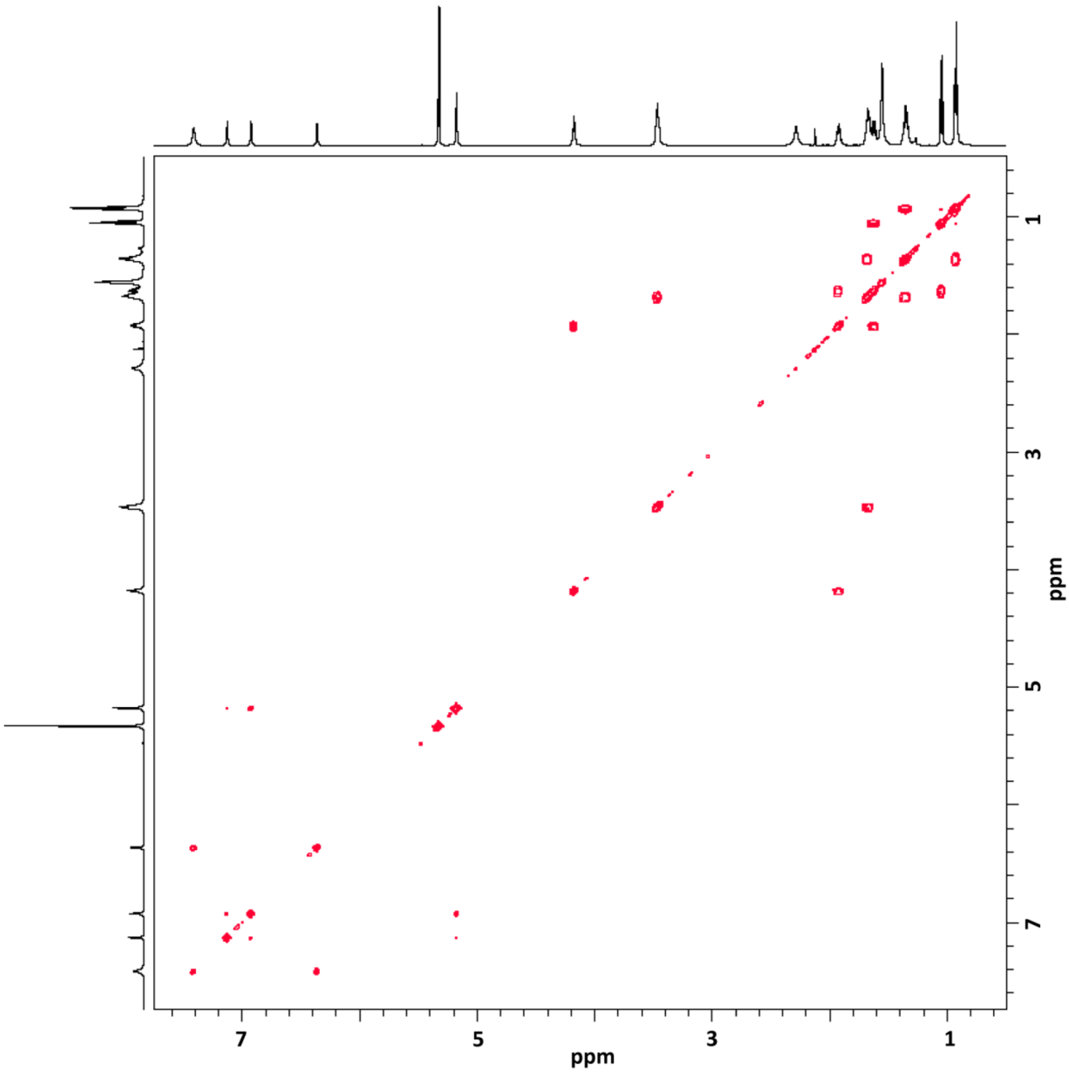

Supplementary Figure 8 | COSY NMR (600 MHz) spectrum of bis(merocyanine) 1 in CD_2Cl_2 (c = 1 x 10^{-3} M) at 295 K.

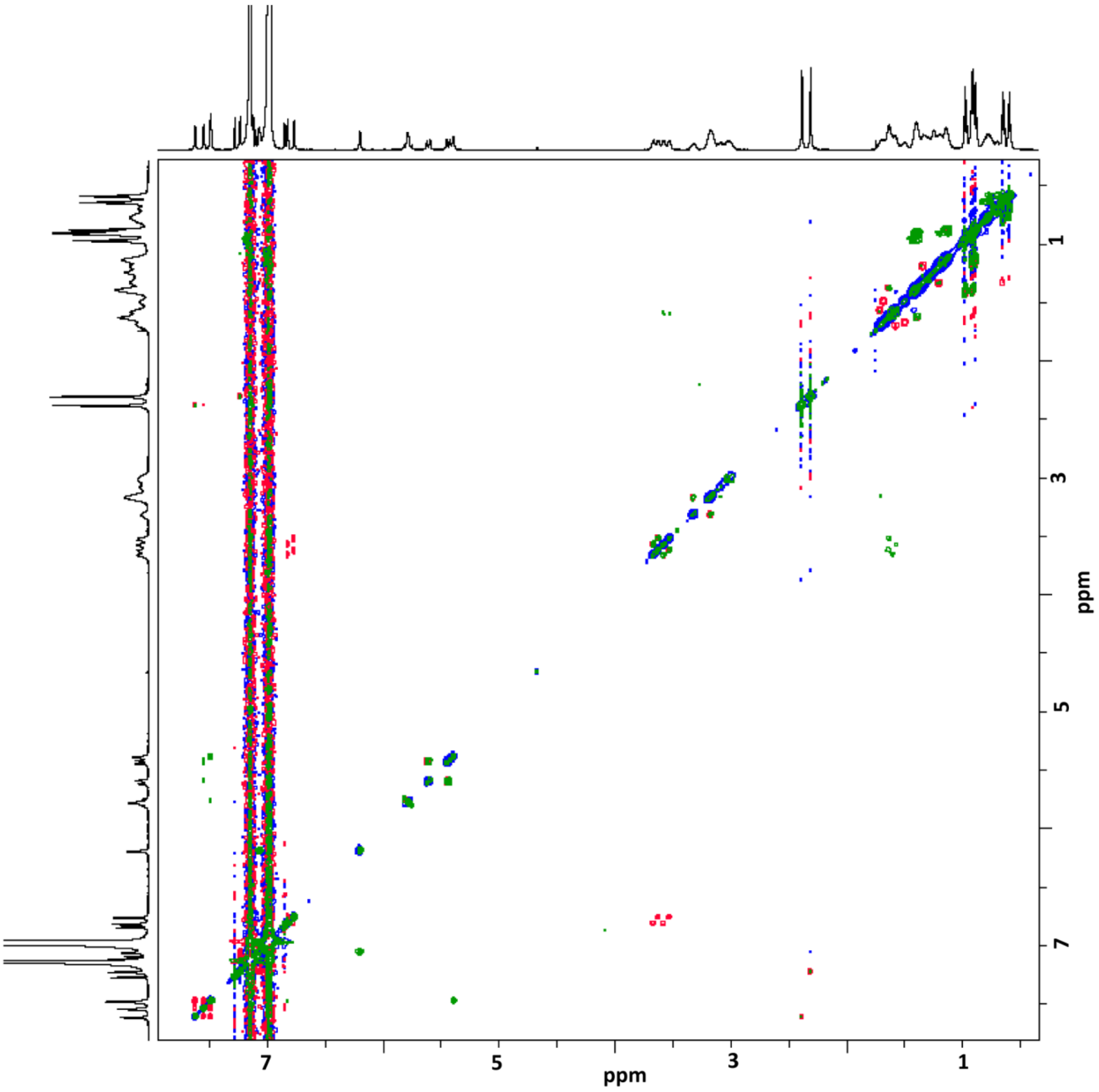

Supplementary Figure 9 | Superposed ROESY NMR and COSY NMR spectra of bis(merocyanine) 1. Entire superposed ROESY (600 MHz; negative signals marked in blue and positive signals in red, respectively) and COSY NMR (600 MHz; marked in green) spectra of bis(merocyanine) **1** in C_6D_5Cl ($c = 1 \times 10^{-3}$ M) at 233 K.

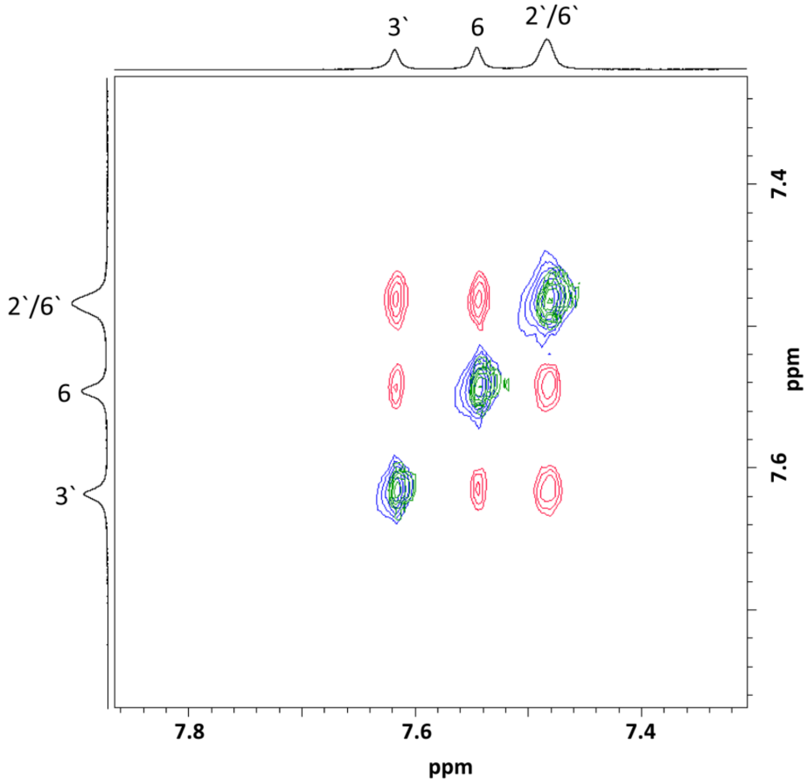

Supplementary Figure 10 | Selected region of superposed ROESY NMR and COSY NMR spectra of bis(merocyanine) 1. Selected region of superposed ROESY NMR (600 MHz; negative signals marked in blue and positive signals in red, respectively) and COSY NMR (600 MHz; marked in green) spectra of bis(merocyanine) **1** in C_6D_5Cl (c = 1 x 10^{-3} M) at 233 K.

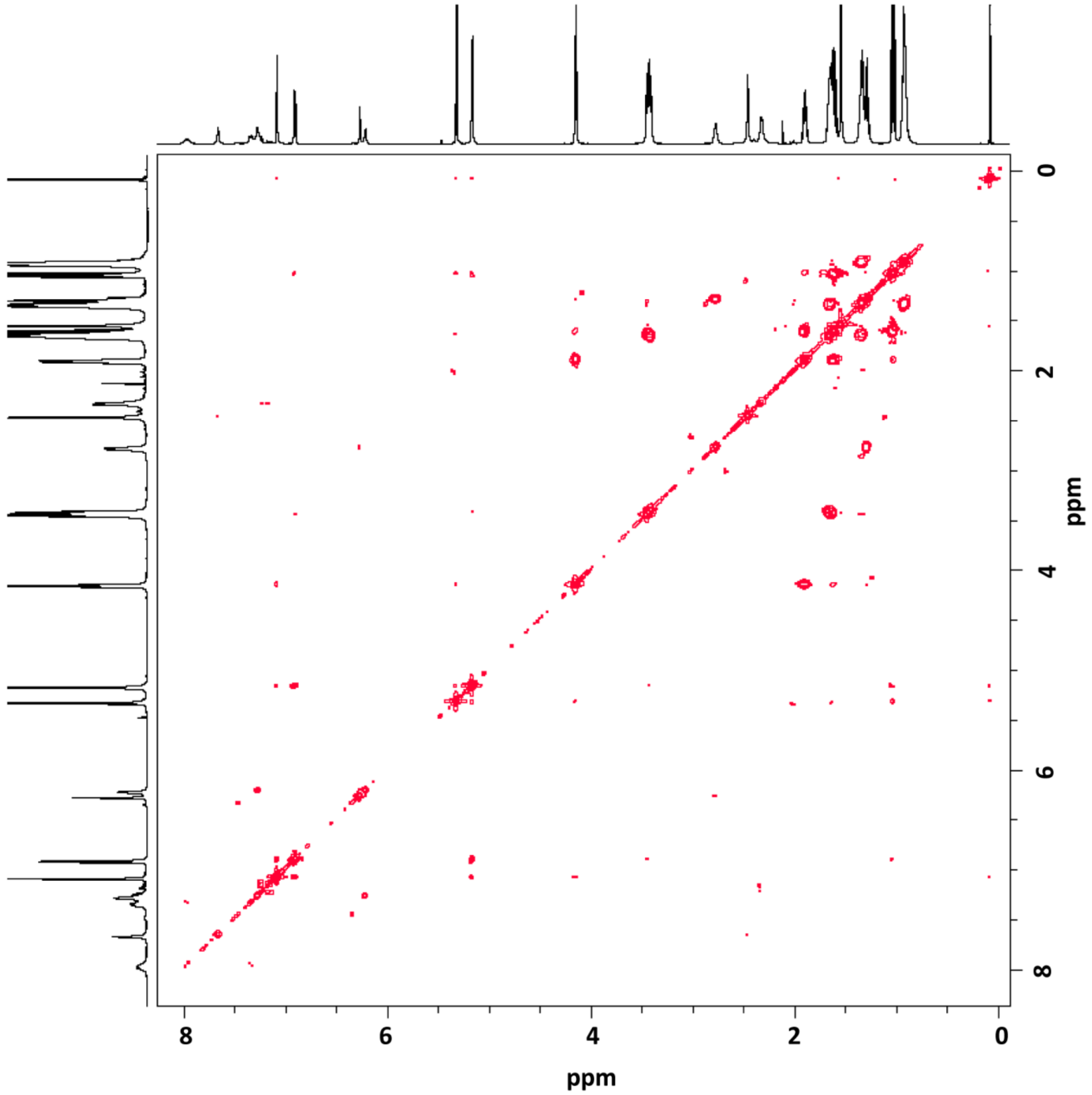

Supplementary Figure 11 | COSY NMR (600 MHz) spectrum of bis(merocyanine) 2 in CD_2Cl_2 (c = 5 x 10^{-3} M) at 295 K.

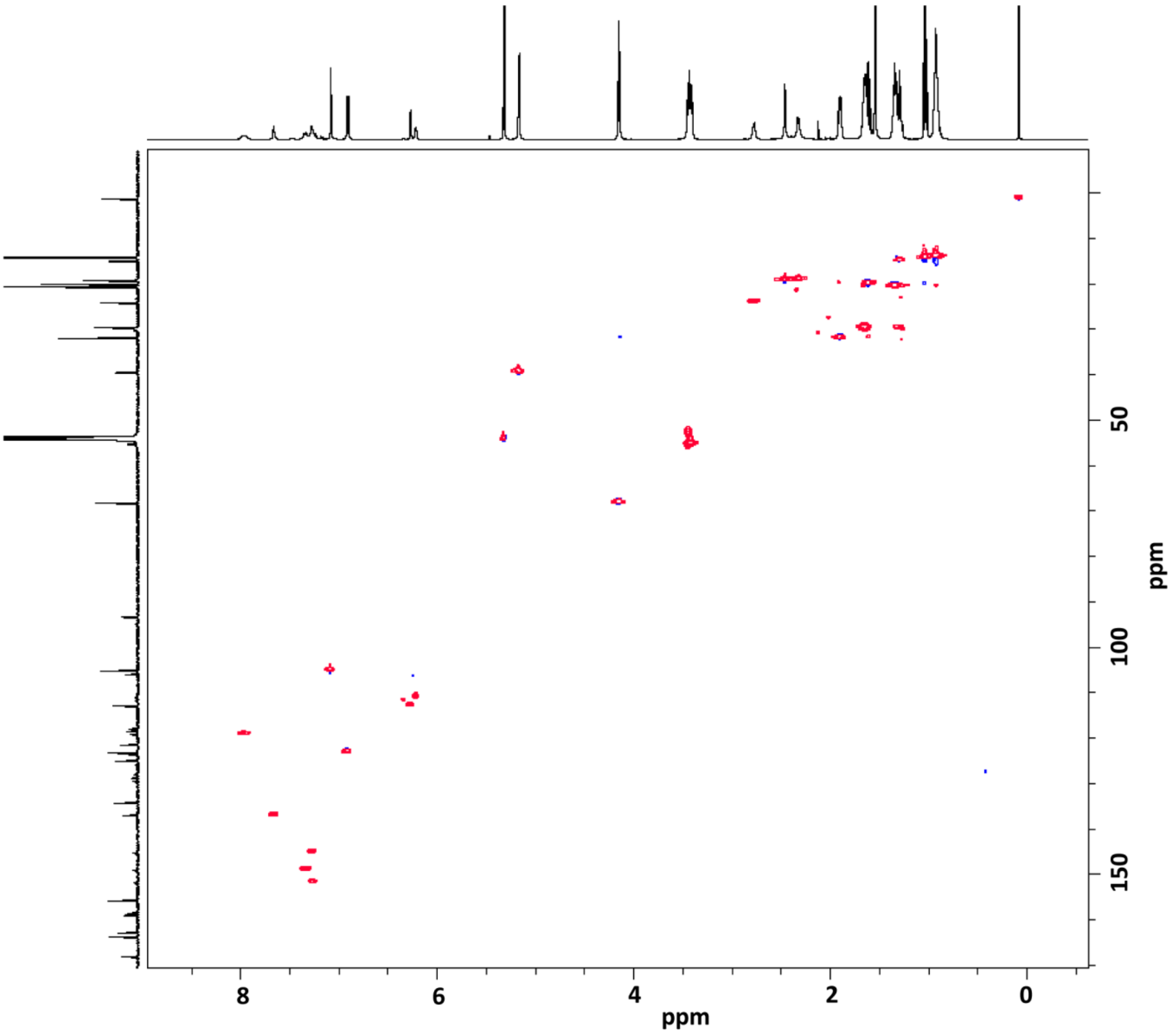

Supplementary Figure 12 | HSQC NMR (600 MHz) spectrum of bis(merocyanine) 2 in CD_2Cl_2 (c = 5 x 10^{-3} M) at 295 K.

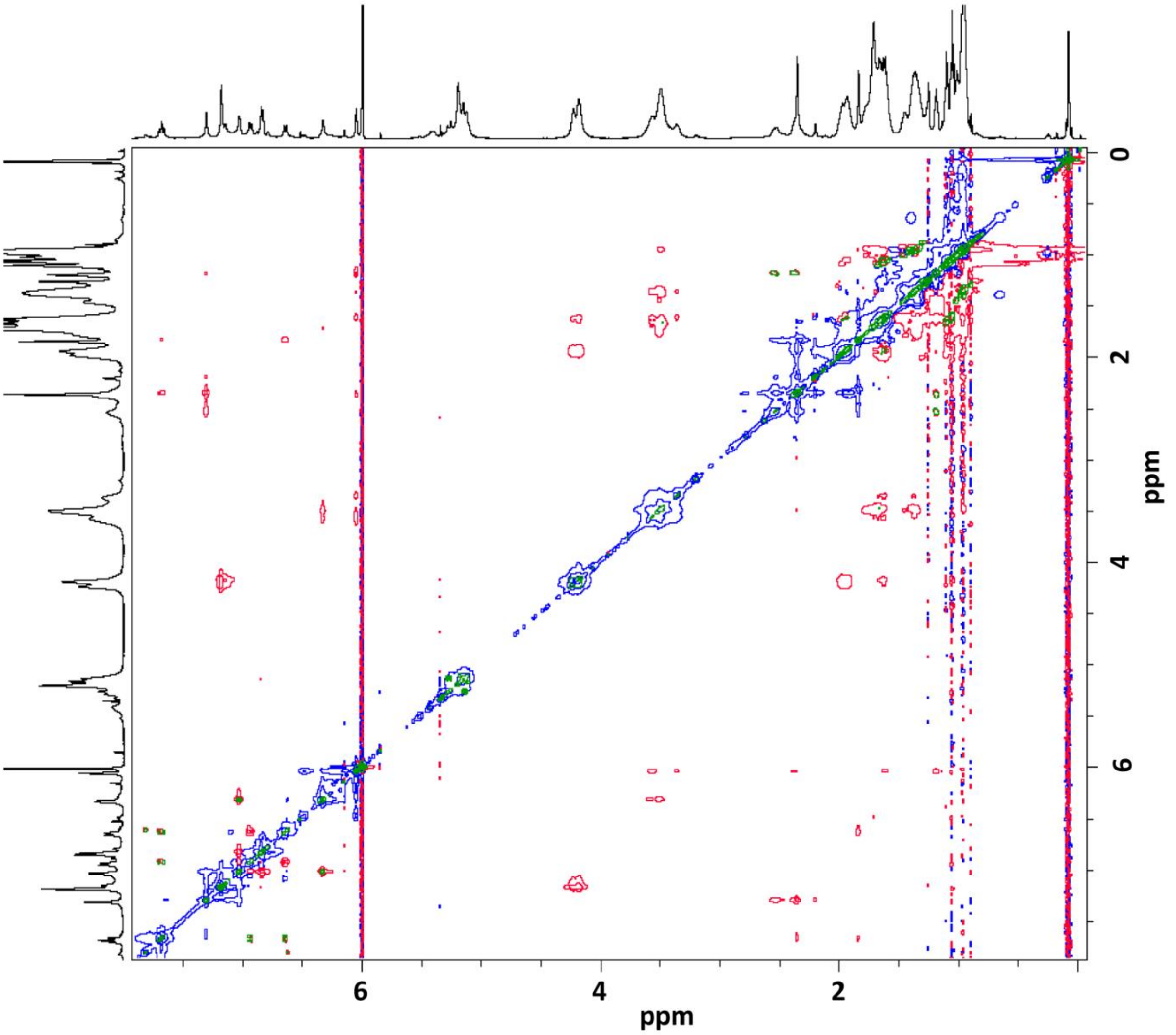

Supplementary Figure 13 | Superposed ROESY NMR and COSY NMR spectra of bis(merocyanine) 2. Superposed ROESY NMR (600 MHz; negative signals marked in blue and positive signals in red, respectively) and COSY NMR (600 MHz; marked in green) spectra of bis(merocyanine) **2** in a 1:1 mixture of 1,1,2,2-tetrachloroethane-d_2/tetrachloromethane (c = 8 x 10^{-3} M) at 253 K.

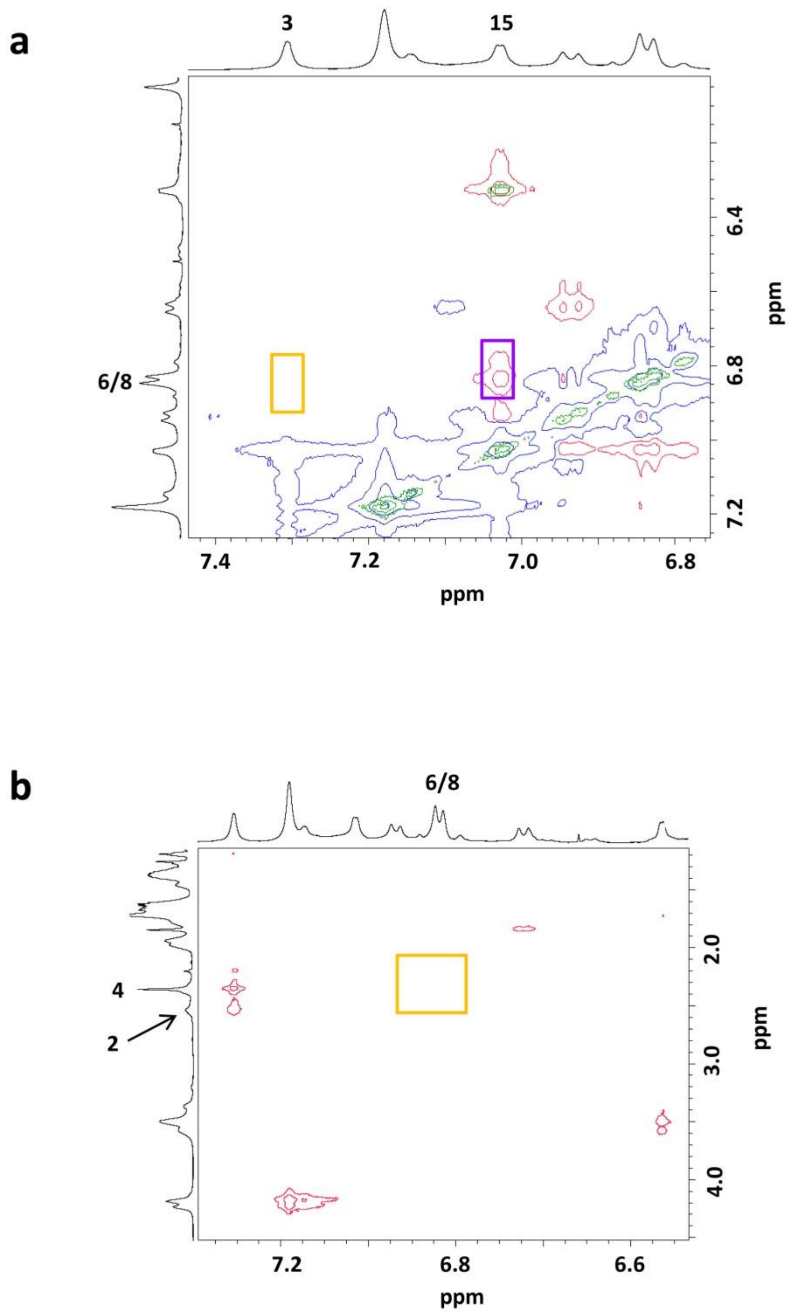

Supplementary Figure 14 | Selected regions of superposed ROESY NMR and COSY NMR spectra of bis(merocyanine) 2. (**a**) and (**b**): Selected regions of superposed ROESY NMR (600 MHz; negative signals marked in blue and positive signals in red, respectively) and COSY NMR (600 MHz; marked in green) spectra of bis(merocyanine) **2** in a 1:1 mixture of 1,1,2,2-tetrachloroethane-d_2/tetrachloromethane ($c = 8 \times 10^{-3}$ M) at 253 K. The orange rectangles indicate the missing cross-signals that should be present if the short chromophores are located at the inner part of the tetrachromophoric stack, while the violet rectangle highlights the cross-peak between the protons of the naphthalene spacer (protons 6 and 8) and the long chromophore (proton 15).

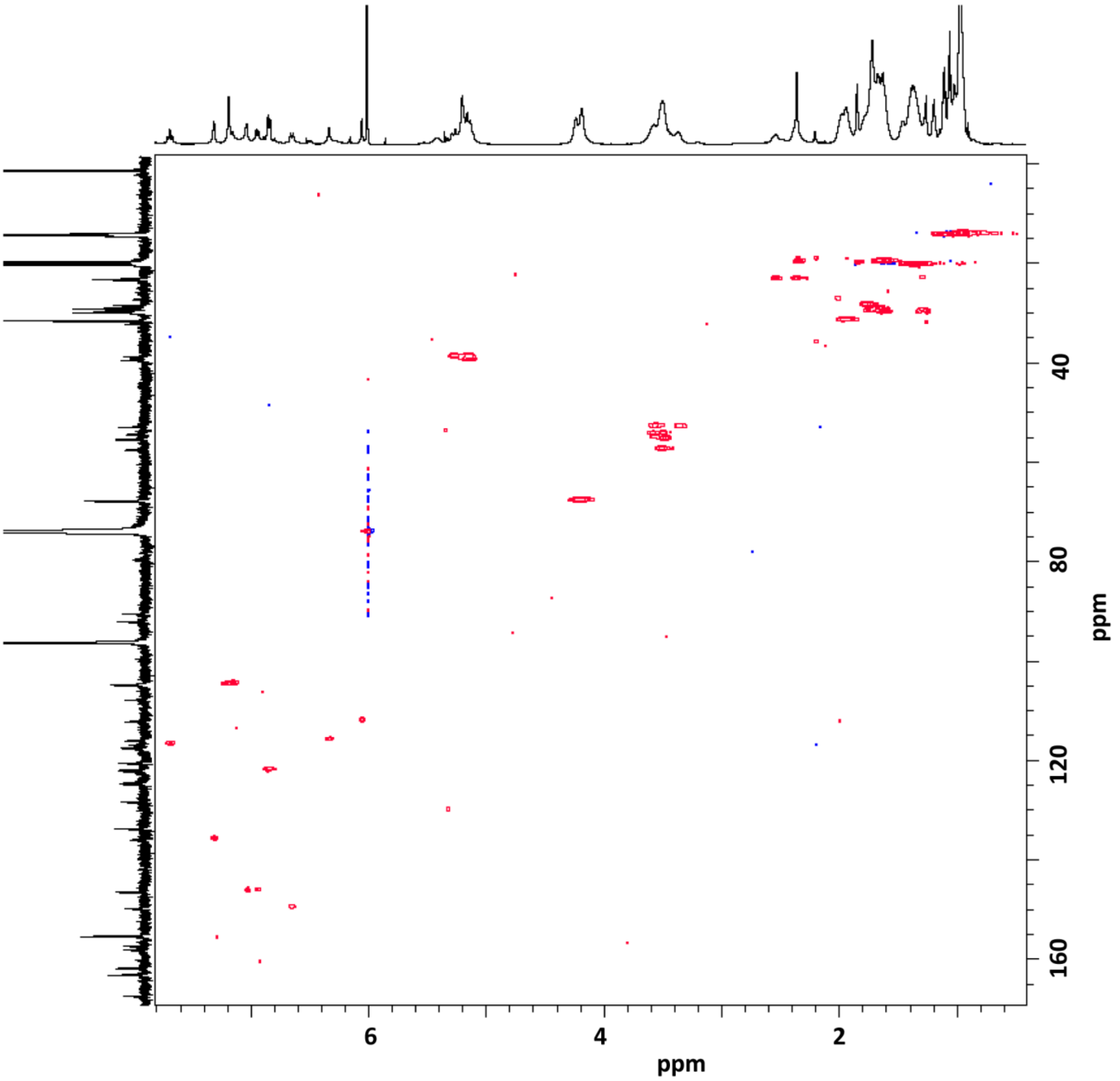

Supplementary Figure 15 | HSQC NMR (600 MHz) spectrum of bis(merocyanine) 2 in a 1:1 mixture of 1,1,2,2-tetrachloroethane-d_2/tetrachloromethane (c = 8 x 10^{-3} M) at 253 K.

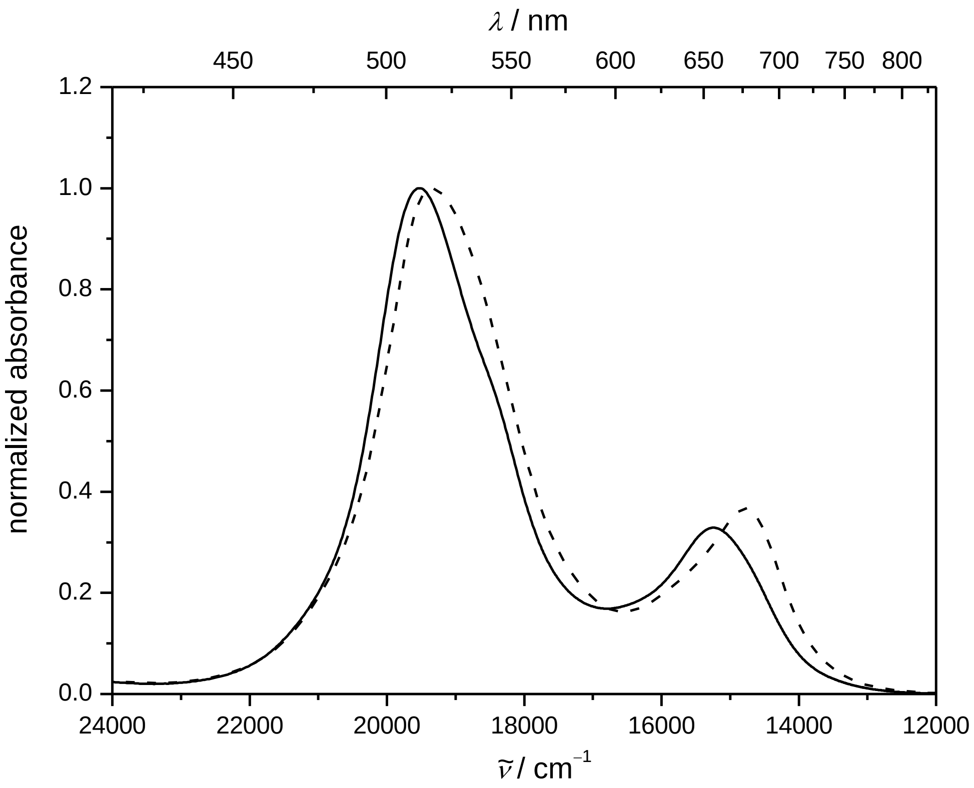

Supplementary Figure 16 | UV/Vis absorption spectra of bis(merocyanine) 2. UV/Vis absorption spectrum of bis(merocyanine) **2** in a 1:1 mixture of tetrachloroethane-d_2/tetrachloromethane (c = 8 x 10^{-3} M, solid line) at 298 K. The ideal dimer spectrum obtained by extrapolation of the data from concentration-dependent UV/Vis spectroscopy in chlorobenzene is shown (dashed line) for comparison.

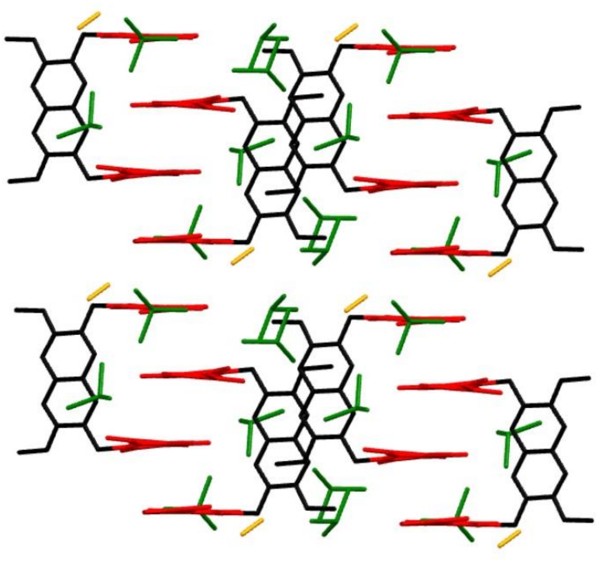

Supplementary Figure 17 | Single crystal X-ray structure of bis(merocyanine) 1. Molecular packing of bis(merocyanine) **1** in the solid state (hydrogen atoms are omitted for clarity and butyl chains were replaced by methyl groups). The chromophores are colored in red and the naphthalene spacer unit in black. Chloroform and methanol solvent molecules are visualized in green and orange, respectively.

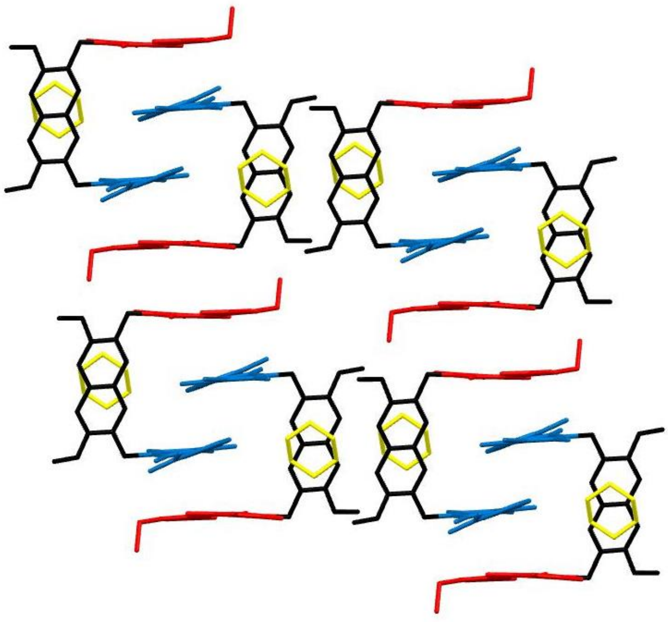

Supplementary Figure 18 | Single crystal X-ray structure of bis(merocyanine) 2. Molecular packing of bis(merocyanine) **2** in the solid state (hydrogen atoms are omitted for clarity and butyl chains were replaced by methyl groups). The chromophores are colored in red (short chromophore) and blue (long chromophore), respectively, while the naphthalene spacer unit is colored in black. Solvent (1,4-dioxane) molecules are colored in yellow.

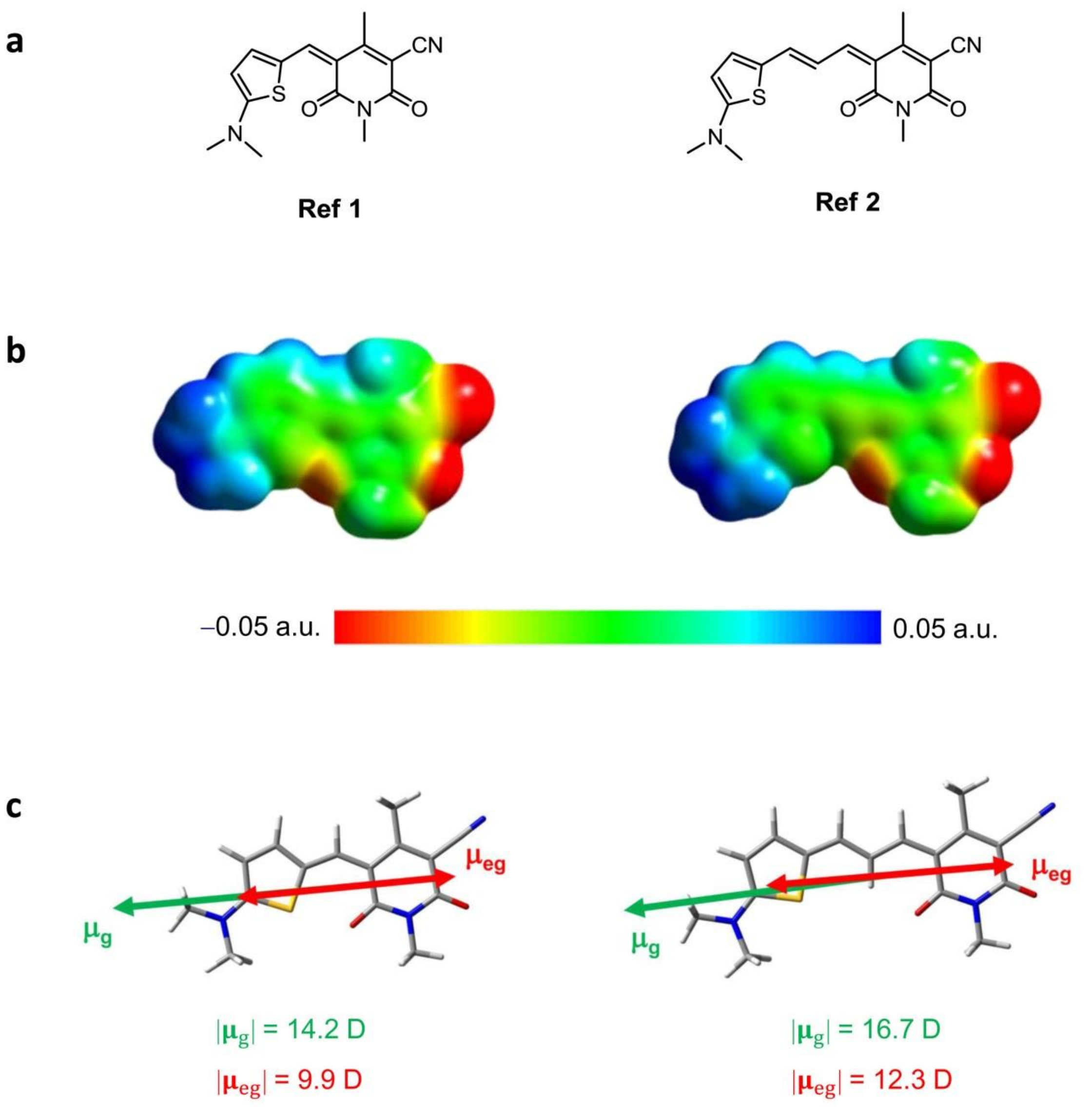

Supplementary Figure 19 | Results of DFT calculations for Ref 1 and Ref 2. (**a**) Chemical structures and (**b**) electrostatic potential surfaces (isovalue = 0.001 a.u.) of geometry-optimized (DFT, B97D3/def2-SVP) merocyanine **Ref 1** (left) and **Ref 2** (right); (**c**) geometry-optimized structures of **Ref 1** (left) and **Ref 2** (right). The green arrows in (**c**) display the ground state dipole moments and the red arrows the transition dipole moments. The latter were obtained by TDDFT calculations (ωB97/def2-SVP).

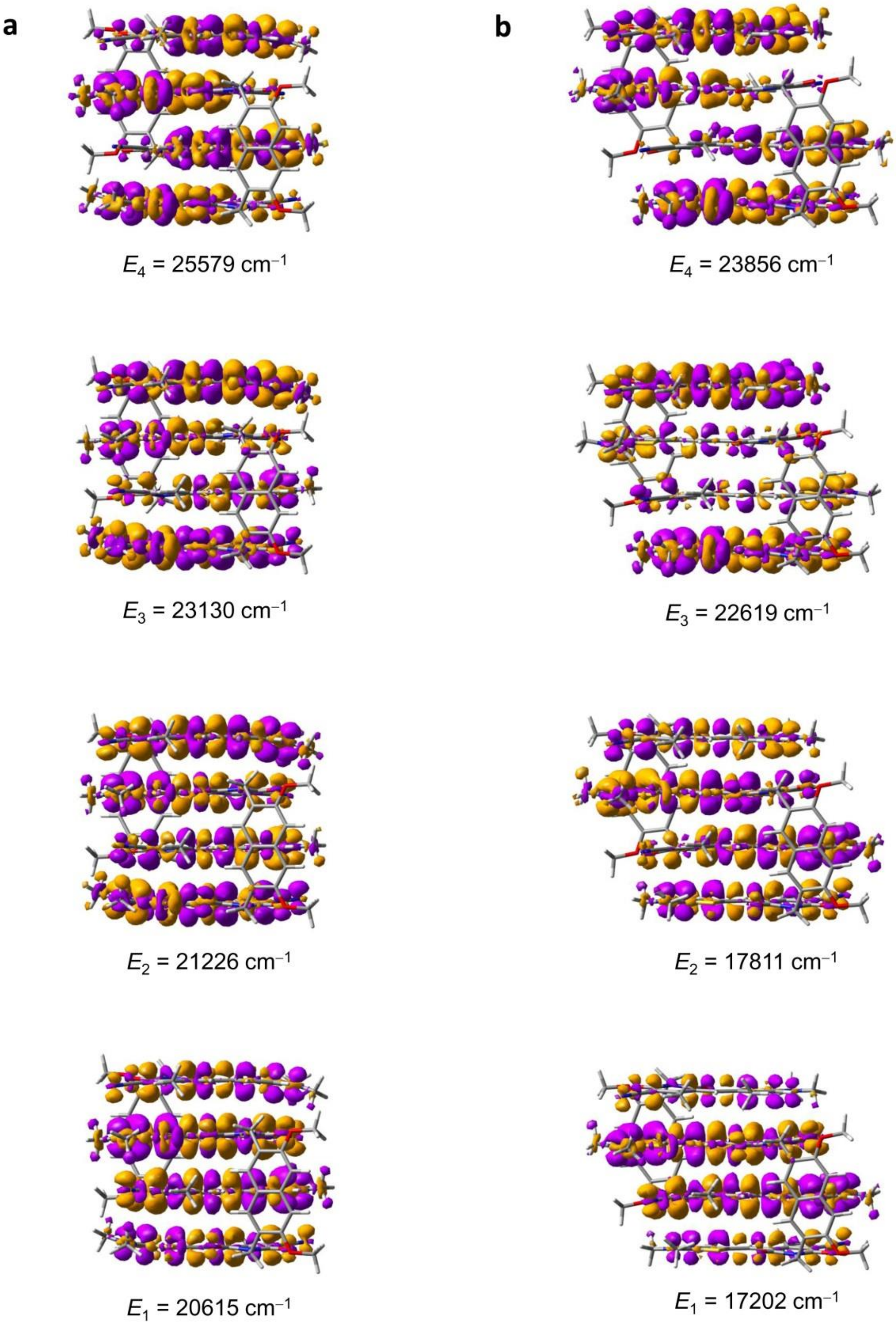

Supplementary Figure 20 | Transition densities (isovalue = 0.0007 a.u.) and energies of the four excited states of the dimer aggregates of bis(merocyanine) dyes (a) 1 and (b) 2 obtained by TDDFT calculations (ωB97/def2-SVP).

Supplementary Table 1 | Summary of ^{1}H NMR (600 MHz) data for bis(merocyanine) 1. Chemical shifts δ (ppm) and coupling constants J (Hz), and assignment of the significant chromophore and backbone proton signals of bis(merocyanine) dye **1** monomer (CD_2Cl_2 at 295 K) and aggregate (C_6D_5Cl at 233 K). The two sets of signals for the aggregate protons are denoted with (outer part of dye stack) and without prime (inner part of dye stack), respectively.

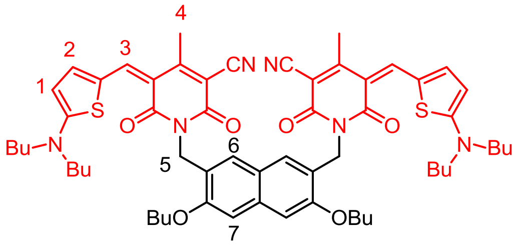

1

proton	δ_H [ppm] in CD_2Cl_2	δ_H [ppm] in C_6D_5Cl
1	6.36 (d, ^{3}J = 5.2 Hz, 2H)	6.19 (d, ^{3}J = 5.0 Hz, 1H)
1’		5.39 (d, ^{3}J = 4.2 Hz, 1H)
2	7.41 (2H, overlapped with proton 3)	7.06 (d, ^{3}J = 5.0 Hz, 1H)
2’		7.48 (2H, overlapped with proton 6’)
3	7.41 (2H, overlapped with proton 2)	7.22 (s, 1H)
3’		7.61 (s, 1H)
4	2.28 (br, 6H)	2.30 (s, 3H)
4’		2.38 (s, 3H)
5α	5.17 (s, 4H)	5.60 (d, ^{2}J = 16.9 Hz, 1H)
5β		5.43 (d, ^{2}J = 15.5 Hz, 1H)
5’α		5.79/5.76 (d, ^{2}J = 15.5 Hz, 1H)
5’β		5.79/5.76 (d, ^{2}J = 15.5 Hz, 1H)
6	6.92 (s, 2H)	7.54 (s, 1H)
6’		7.48 (overlapped with proton 2’, 1H)
7	7.12 (s, 2H)	6.76 (s, 1H)
7’		6.81 (s, 1H)

Supplementary Table 2 | Summary of ^{1}H NMR (600 MHz) data for bis(merocyanine) 2. Chemical shifts δ (ppm) and coupling constants J (Hz), and assignment of the significant chromophore and backbone proton signals of bis(merocyanine) dye **2** monomer (CD_2Cl_2 at 295 K) and aggregate (1:1 mixture of 1,1,2,2-tetrachloroethane-d_2/tetrachloromethane at 253 K).

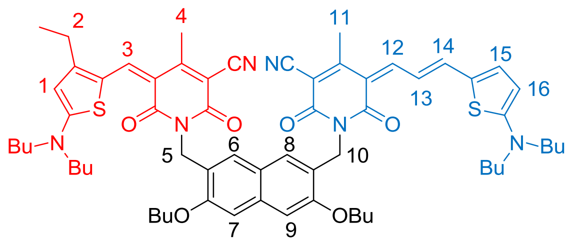

2

proton	δ_H [ppm] in CD_2Cl_2	δ_H [ppm] in 1,1,2,2-tetrachloroethane-d_2/CCl_4 1:1
1	6.27 (s, 1H)	6.05 (s, 1H)
2	2.77 (q, ^3J = 7.1 Hz, 2H)	2.53 (br 2H)
3	7.69 (s, 1H)	7.31 (s, 1H)
4	2.46 (s, 3H)	2.35 (s, 3H)
5	5.17/5.16 (s, 2H, could not be distinguished from protons 10)	5.33-5.08 (br, 2H, overlapped with protons 10)
6	6.91/6.90 (s, 1H, could not be distinguished from proton 8)	6.85/6.83 (s, 1H, could not be distinguished from proton 8)
7	7.08 (s, 1H, overlapped with proton 9)	7.18/7.15 (s, 1H, could not be distinguished from proton 9)
8	6.91/6.90 (s, 1H, could not be distinguished from proton 6)	6.85/6.83 (s, 1H, could not be distinguished from proton)
9	7.08 (s, 1H, overlapped with proton 7)	7.18/7.15 (s, 1H, could not be distinguished from proton 7)
10	5.17/5.16 (s, 2H, could not be distinguished from proton 5)	5.33-5.08 (br, 2H, overlapped with protons 5)
11	2.32 (s, 3H)	1.84 (s, 3H)
12	7.27 (1H, overlapped with proton 15)	6.64 (d, ^3J = 13.0 Hz, 1H)
13	7.96 (br, 1H)	7.68 (dd, ^3J = 13.0 Hz, ^3J = 12.0 Hz, 1H)
14	7.34 (d, ^3J = 12.5 Hz, 1H)	6.94 (d, ^3J = 12.0 Hz, 1H)
15	7.27 (1H, overlap with proton 12)	7.03 (d, ^3J = 4.1 Hz, 1H)
16	6.21 (d, ^3J = 3.3 Hz, 1H)	6.33 (d, ^3J = 4.1 Hz, 1H)

Supplementary Table 3 | Results of TDDFT calculations for bis(merocyanine) dyes 1 and 2. Excited state energies and corresponding oscillator strengths (*f*) of the dimers of bis(merocyanine) **1** and **2** obtained by TDDFT calculations (ωB97/def2-SVP).

	bis(merocyanine) **1**	bis(merocyanine) **2**
excited state energy (oscillator strength)	20615 cm^{-1} (f = 0)	17202 cm^{-1} (f = 0)
	21226 cm^{-1} (f = 0.0614)	17811 cm^{-1} (f = 0.3213)
	23130 cm^{-1} (f = 0)	22619 cm^{-1} (f = 0)
	25579 cm^{-1} (f = 2.4697)	23856 cm^{-1} (f = 2.6982)

Supplementary Table 4 | Transition charges for Ref 1. Cartesian coordinates of the geometry optimized structure (B97D3/def-2SVP) of **Ref 1** and transition charges derived from Mulliken population analysis of the transition density obtained by TDDFT calculations (ωB97/def2-SVP).

	x	*y*	*z*	transition charges
C	3.77493	−0.33666	−0.03340	−0.03717
C	3.62867	−1.74358	−0.01579	−0.00131
C	2.28565	−2.12202	−0.00724	−0.04826
C	1.35256	−1.05516	−0.00554	0.01729
S	2.22376	0.49426	−0.01089	−0.03900
N	4.93693	0.36561	−0.06159	−0.02846
C	6.20091	−0.35028	0.04026	0.02470
C	4.90879	1.81896	0.03031	0.02014
C	−0.03750	−1.30055	0.00251	−0.02255
C	−1.21235	−0.53444	0.00413	0.04969
C	−2.49860	−1.19618	0.00616	−0.01850
C	−3.65940	−0.43258	0.00283	0.05807
C	−3.64949	1.04376	−0.00266	0.01565
N	−2.36755	1.63814	−0.00180	−0.00750
C	−1.15869	0.93866	0.00399	0.01046
O	−0.10191	1.57938	0.00938	0.05791
C	−2.28036	3.09972	−0.00375	−0.00898
O	−4.66913	1.72142	−0.00703	0.03466
C	−2.59061	−2.70191	0.01082	0.00036
C	−4.95384	−1.03821	0.00396	0.00826
N	−5.99935	−1.56823	0.00510	0.04140
H	4.46361	−2.44712	−0.01034	−0.02142
H	1.95108	−3.16493	−0.00021	−0.02890
H	7.03167	0.35955	−0.08894	−0.01999
H	6.27914	−1.11957	−0.74978	−0.02303
H	6.31393	−0.85084	1.02311	−0.02787
H	4.61016	2.16494	1.04112	−0.02983
H	5.90608	2.22141	−0.20378	−0.01680
H	−0.21542	−2.38199	0.00721	0.00525
H	−3.30537	3.49270	−0.01038	0.00517
H	−1.72528	3.44255	−0.89276	0.01419
H	−1.73587	3.44587	0.89056	0.01409
H	−3.63769	−3.03805	0.01463	0.00084
H	−2.09712	−3.13193	−0.87975	0.01375
H	−2.09207	−3.12678	0.90102	0.01369
H	4.18720	2.23386	−0.69769	−0.02599

Supplementary Table 5 | Transition charges for Ref 2. Cartesian coordinates of the geometry optimized structure (B97D3/def-2SVP) of **Ref 2** and transition charges derived from Mulliken population analysis of the transition density obtained by TDDFT calculations (ωB97/def2-SVP).

	x	*y*	*z*	transition charges
C	4.99540	−0.13305	−0.03564	0.03473
C	5.11274	−1.53791	−0.02397	−0.00434
C	3.86204	−2.18061	−0.02329	0.05378
C	2.75770	−1.31429	−0.02112	−0.04032
S	3.31178	0.36976	−0.01624	0.05005
N	5.99484	0.79056	−0.05877	0.02302
C	7.37109	0.33204	0.06161	−0.02185
C	5.68276	2.20526	0.07924	−0.01917
C	1.38242	−1.65110	−0.01527	0.05935
C	−2.20969	−0.46645	−0.00317	−0.04768
C	−3.48966	−1.13852	0.01098	0.01670
C	−4.65824	−0.38828	0.01287	−0.05147
C	−4.66472	1.08961	0.00074	−0.01415
N	−3.39445	1.69999	−0.01262	0.00605
C	−2.16484	1.02222	−0.01537	−0.01160
O	−1.12665	1.68430	−0.02769	−0.04218
O	−5.69674	1.74979	0.00219	−0.03255
C	−3.56356	−2.64541	0.02386	0.00094
C	−5.94587	−1.00790	0.02682	−0.00645
N	−6.98511	−1.55017	0.03851	−0.03947
H	6.06938	−2.06342	−0.01581	0.02211
H	3.73706	−3.26840	−0.02111	0.02930
H	8.05054	1.18713	−0.07248	0.01935
H	7.60113	−0.41594	−0.71896	0.01974
H	7.57019	−0.12735	1.05140	0.02488
H	5.35328	2.46341	1.10717	0.02688
H	6.57281	2.80356	−0.16901	0.01735
H	1.17148	−2.73151	−0.00735	0.01283
H	−4.60632	−2.99460	0.03361	−0.00439
H	−3.06667	−3.07315	−0.86572	−0.01356
H	−3.05478	−3.05835	0.91370	−0.01358
H	4.87431	2.48733	−0.62064	0.02201
C	−3.32918	3.16252	−0.02502	0.00936
H	−2.77743	3.52229	0.85943	−0.01415
H	−4.35941	3.54145	−0.02093	−0.00747
H	0.49273	0.31761	−0.02713	−0.00899
C	0.31686	−0.76252	−0.01692	−0.04938
H	−1.13981	−2.30422	0.00397	−0.01283
C	−1.02307	−1.21314	−0.00522	0.02123
H	−2.78989	3.50771	−0.92287	−0.01405

Supplementary Table 6 | Exciton coupling energies *J* of the relevant chromophore pairs in the dye stacks of bis(merocyanine) dyes 1 and 2 determined by transition charge method.

	Bis(merocyanine) **1**	Bis(merocyanine) **2**
J_{12}	1576 cm^{-1}	1861 cm^{-1}
J_{23}	1452 cm^{-1}	1108 cm^{-1}

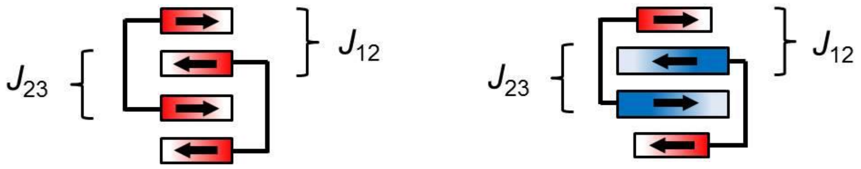

Supplementary Table 7 | Excited state energies of the dimers of bis(merocyanine) 1 and 2 determined on the basis of the eigenvalues of the corresponding Hamiltonian matrix.

	bis(merocyanine) **1**[a]	bis(merocyanine) **2**[b]
E_1	16044 cm^{-1}	13277 cm^{-1}
E_2	17496 cm^{-1}	15090 cm^{-1}
E_3	19514 cm^{-1}	18929 cm^{-1}
E_4	20966 cm^{-1}	19333 cm^{-1}

[a] Calculated from E_E + 18505 cm^{-1}. [b] Calculated from E_E + 16657 cm^{-1}. E_E represents the corresponding eigenvalue of the Hamiltonian matrix. The translation is necessary due to the definition of the zero level.

Supplementary Table 8 | Calculated coefficients of the normed eigenvectors of the Hamiltonian matrix of the quadruple dye stack of bis(merocyanine) 1.

	c_1	c_2	c_3	c_4
E_{E1}	–0.3813	0.5954	–0.5954	0.3813
E_{E2}	0.5955	–0.3813	–0.3813	0.5955
E_{E3}	–0.5955	–0.3813	0.3813	0.5955
E_{E4}	0.3813	0.5954	0.5954	0.3813

Supplementary Table 9 | Calculated coefficients of the normed eigenvectors of the Hamiltonian matrix of the quadruple dye stack of bis(merocyanine) 2.

	c_1	c_2	c_3	c_4
E_{E1}	–0.2487	0.6619	–0.6619	0.2487
E_{E2}	0.3605	–0.6084	–0.6084	0.3605
E_{E3}	–0.6619	–0.2486	0.2486	0.6619
E_{E4}	0.6083	0.3604	0.3604	0.6083

Supplementary Note 1 | Components of the normed eigenvectors of the Hamiltonian matrix for the dye stack of bis(merocyanine) 1.

Excited state 1:

$$c_1 = -\frac{1}{\sqrt{2+\frac{\left(J_{23}+\sqrt{4J_{12}^2+J_{23}^2}\right)^2}{2J_{12}^2}}}$$

$$c_2 = \frac{J_{23}+\sqrt{4J_{12}^2+J_{23}^2}}{2J_{12}\sqrt{2+\frac{\left(J_{23}+\sqrt{4J_{12}^2+J_{23}^2}\right)^2}{2J_{12}^2}}}$$

$$c_3 = -\frac{J_{23}+\sqrt{4J_{12}^2+J_{23}^2}}{2J_{12}\sqrt{2+\frac{\left(J_{23}+\sqrt{4J_{12}^2+J_{23}^2}\right)^2}{2J_{12}^2}}}$$

$$c_4 = \frac{1}{\sqrt{2+\frac{\left(J_{23}+\sqrt{4J_{12}^2+J_{23}^2}\right)^2}{2J_{12}^2}}}$$

Excited state 2:

$$c_1 = \frac{1}{\sqrt{2+\frac{\left(J_{23}-\sqrt{4J_{12}^2+J_{23}^2}\right)^2}{2J_{12}^2}}}$$

$$c_2 = \frac{J_{23}-\sqrt{4J_{12}^2+J_{23}^2}}{2J_{12}\sqrt{2+\frac{\left(J_{23}-\sqrt{4J_{12}^2+J_{23}^2}\right)^2}{2J_{12}^2}}}$$

$$c_3 = \frac{J_{23}-\sqrt{4J_{12}^2+J_{23}^2}}{2J_{12}\sqrt{2+\frac{\left(J_{23}-\sqrt{4J_{12}^2+J_{23}^2}\right)^2}{2J_{12}^2}}}$$

$$c_4 = \frac{1}{\sqrt{2+\frac{\left(J_{23}-\sqrt{4J_{12}^2+J_{23}^2}\right)^2}{2J_{12}^2}}}$$

Excited state 3:

$$c_1 = -\frac{1}{\sqrt{2+\frac{\left(J_{23}-\sqrt{4J_{12}^2+J_{23}^2}\right)^2}{2J_{12}^2}}}$$

$$c_2 = \frac{J_{23}-\sqrt{4J_{12}^2+J_{23}^2}}{2J_{12}\sqrt{2+\frac{\left(J_{23}-\sqrt{4J_{12}^2+J_{23}^2}\right)^2}{2J_{12}^2}}}$$

$$c_3 = -\frac{J_{23}-\sqrt{4J_{12}^2+J_{23}^2}}{2J_{12}\sqrt{2+\frac{\left(J_{23}-\sqrt{4J_{12}^2+J_{23}^2}\right)^2}{2J_{12}^2}}}$$

$$c_4 = \frac{1}{\sqrt{2+\frac{\left(J_{23}-\sqrt{4J_{12}^2+J_{23}^2}\right)^2}{2J_{12}^2}}}$$

Excited state 4:

$$c_1 = \frac{1}{\sqrt{2+\frac{\left(J_{23}+\sqrt{4J_{12}^2+J_{23}^2}\right)^2}{2J_{12}^2}}}$$

$$c_2 = \frac{J_{23}+\sqrt{4J_{12}^2+J_{23}^2}}{2J_{12}\sqrt{2+\frac{\left(J_{23}+\sqrt{4J_{12}^2+J_{23}^2}\right)^2}{2J_{12}^2}}}$$

$$c_3 = \frac{J_{23}+\sqrt{4J_{12}^2+J_{23}^2}}{2J_{12}\sqrt{2+\frac{\left(J_{23}+\sqrt{4J_{12}^2+J_{23}^2}\right)^2}{2J_{12}^2}}}$$

$$c_4 = \frac{1}{\sqrt{2+\frac{\left(J_{23}+\sqrt{4J_{12}^2+J_{23}^2}\right)^2}{2J_{12}^2}}}$$

Supplementary Note 2 | Components of the normed eigenvectors of the Hamiltonian matrix for the dye stack of bis(merocyanine) 2.

Excited state 1:

$$c_1 = -\frac{1}{\sqrt{2+\frac{\left(2\Delta E+J_{23}+\sqrt{4\Delta E^2+4J_{12}^2+4\Delta EJ_{23}+J_{23}^2}\right)^2}{2J_{12}^2}}}$$

$$c_2 = \frac{2\Delta E+J_{23}+\sqrt{4\Delta E^2+4J_{12}^2+4\Delta EJ_{23}+J_{23}^2}}{2J_{12}\sqrt{2+\frac{\left(2\Delta E+J_{23}+\sqrt{4\Delta E^2+4J_{12}^2+4\Delta EJ_{23}+J_{23}^2}\right)^2}{2J_{12}^2}}}$$

$$c_3 = -\frac{2\Delta E+J_{23}+\sqrt{4\Delta E^2+4J_{12}^2+4\Delta EJ_{23}+J_{23}^2}}{2J_{12}\sqrt{2+\frac{\left(2\Delta E+J_{23}+\sqrt{4\Delta E^2+4J_{12}^2+4\Delta EJ_{23}+J_{23}^2}\right)^2}{2J_{12}^2}}}$$

$$c_4 = \frac{1}{\sqrt{2+\frac{\left(2\Delta E+J_{23}+\sqrt{4\Delta E^2+4J_{12}^2+4\Delta EJ_{23}+J_{23}^2}\right)^2}{2J_{12}^2}}}$$

Excited state 2:

$$c_1 = \frac{1}{\sqrt{2+\frac{\left(2\Delta E-J_{23}+\sqrt{4\Delta E^2+4J_{12}^2-4\Delta EJ_{23}+J_{23}^2}\right)^2}{2J_{12}^2}}}$$

$$c_2 = -\frac{2\Delta E-J_{23}+\sqrt{4\Delta E^2+4J_{12}^2-4\Delta EJ_{23}+J_{23}^2}}{2J_{12}\sqrt{2+\frac{\left(2\Delta E-J_{23}+\sqrt{4\Delta E^2+4J_{12}^2-4\Delta EJ_{23}+J_{23}^2}\right)^2}{2J_{12}^2}}}$$

$$c_3 = -\frac{2\Delta E - J_{23} + \sqrt{4\Delta E^2 + 4J_{12}^2 - 4\Delta E J_{23} + J_{23}^2}}{2J_{12}\sqrt{2 + \frac{\left(2\Delta E - J_{23} + \sqrt{4\Delta E^2 + 4J_{12}^2 - 4\Delta E J_{23} + J_{23}^2}\right)^2}{2J_{12}^2}}}$$

$$c_4 = \frac{1}{\sqrt{2 + \frac{\left(2\Delta E - J_{23} + \sqrt{4\Delta E^2 + 4J_{12}^2 - 4\Delta E J_{23} + J_{23}^2}\right)^2}{2J_{12}^2}}}$$

Excited state 3:

$$c_1 = -\frac{1}{\sqrt{2 + \frac{\left(2\Delta E + J_{23} - \sqrt{4\Delta E^2 + 4J_{12}^2 + 4\Delta E J_{23} + J_{23}^2}\right)^2}{2J_{12}^2}}}$$

$$c_2 = \frac{2\Delta E + J_{23} - \sqrt{4\Delta E^2 + 4J_{12}^2 + 4\Delta E J_{23} + J_{23}^2}}{2J_{12}\sqrt{2 + \frac{\left(2\Delta E + J_{23} - \sqrt{4\Delta E^2 + 4J_{12}^2 + 4\Delta E J_{23} + J_{23}^2}\right)^2}{2J_{12}^2}}}$$

$$c_3 = -\frac{2\Delta E + J_{23} - \sqrt{4\Delta E^2 + 4J_{12}^2 + 4\Delta E J_{23} + J_{23}^2}}{2J_{12}\sqrt{2 + \frac{\left(2\Delta E + J_{23} - \sqrt{4\Delta E^2 + 4J_{12}^2 + 4\Delta E J_{23} + J_{23}^2}\right)^2}{2J_{12}^2}}}$$

$$c_4 = \frac{1}{\sqrt{2 + \frac{\left(2\Delta E + J_{23} - \sqrt{4\Delta E^2 + 4J_{12}^2 + 4\Delta E J_{23} + J_{23}^2}\right)^2}{2J_{12}^2}}}$$

Excited state 4:

$$c_1 = \frac{1}{\sqrt{2 + \frac{\left(2\Delta E - J_{23} - \sqrt{4\Delta E^2 + 4J_{12}^2 - 4\Delta E J_{23} + J_{23}^2}\right)^2}{2J_{12}^2}}}$$

$$c_2 = -\frac{2\Delta E - J_{23} - \sqrt{4\Delta E^2 + 4J_{12}^2 - 4\Delta E J_{23} + J_{23}^2}}{2J_{12}\sqrt{2 + \frac{\left(2\Delta E - J_{23} - \sqrt{4\Delta E^2 + 4J_{12}^2 - 4\Delta E J_{23} + J_{23}^2}\right)^2}{2J_{12}^2}}}$$

$$c_3 = -\frac{2\Delta E - J_{23} - \sqrt{4\Delta E^2 + 4J_{12}^2 - 4\Delta E J_{23} + J_{23}^2}}{2J_{12}\sqrt{2 + \frac{\left(2\Delta E - J_{23} - \sqrt{4\Delta E^2 + 4J_{12}^2 - 4\Delta E J_{23} + J_{23}^2}\right)^2}{2J_{12}^2}}}$$

$$c_4 = \frac{1}{\sqrt{2 + \frac{\left(2\Delta E - J_{23} - \sqrt{4\Delta E^2 + 4J_{12}^2 - 4\Delta E J_{23} + J_{23}^2}\right)^2}{2J_{12}^2}}}$$

Supplementary Discussion

The excited state wave function Φ_E of an aggregate consisting of four chromophores can be described as follows:

$$\Phi_E = c_1.\varphi^*_1\ \varphi_2\ \varphi_3\ \varphi_4 + c_2.\varphi_1\ \varphi^*_2\ \varphi_3\ \varphi_4 + c_3.\varphi_1\ \varphi_2\ \varphi^*_3\ \varphi_4 + c_4.\varphi_1\ \varphi_2\ \varphi_3\ \varphi^*_4 \qquad (1)$$

where φ_x (x = 1, 2, 3 or 4) is the ground state wave function of chromophore x and φ^*_x the excited state wave function of the corresponding chromophore. Furthermore, c_1, c_2, c_3 and c_4 represent coefficients that have to be determined. The excited state energy E_E is obtained by applying the Hamiltonian operator $\hat{H}$ on the excited state wave function Φ_E:

$$\hat{H}\,\Phi_E = E_E \Phi_E \qquad (2)$$

Multiplying both sides of Supplementary Equation 2 by $\varphi^*_1\varphi_2\varphi_3\varphi_4$ and integration over the coordinates of the chromophores 1, 2, 3 and 4 (e.g. τ_1, τ_2, τ_3 and τ_4), we obtain with Supplementary Equation 1 the Supplementary Equation 3:

$$c_1 H_{11} + c_2 H_{12} + c_3 H_{13} + c_4 H_{14} = E_E\,(c_1 S_{11} + c_2 S_{12} + c_3 S_{13} + c_4 S_{14}) \qquad (3)$$

with

$H_{11} = \iiiint \varphi^*_1\ \varphi_2\ \varphi_3\ \varphi_4\ \hat{H}\ \varphi^*_1\ \varphi_2\ \varphi_3\ \varphi_4 d\tau_1\ d\tau_2\ d\tau_3\ d\tau_4$

$H_{12} = \iiiint \varphi^*_1\ \varphi_2\ \varphi_3\ \varphi_4\ \hat{H}\ \varphi_1\ \varphi^*_2\ \varphi_3\ \varphi_4 d\tau_1\ d\tau_2\ d\tau_3\ d\tau_4$ etc.

and

$S_{11} = \iiiint \varphi^*_1\ \varphi_2\ \varphi_3\ \varphi_4\ \varphi^*_1\ \varphi_2\ \varphi_3\ \varphi_4 d\tau_1\ d\tau_2\ d\tau_3\ d\tau_4$

$S_{12} = \iiiint \varphi^*_1\ \varphi_2\ \varphi_3\ \varphi_4\ \varphi_1\ \varphi^*_2\ \varphi_3\ \varphi_4 d\tau_1\ d\tau_2\ d\tau_3\ d\tau_4$etc.

Due to orthonormalization of the wave functions, the integrals S simplify to:

$$S_{xy} = 1 \text{ for } x = y$$

$$\text{and } S_{xy} = 0 \text{ for } x \neq y$$

Thus, one obtains for Supplementary Equation 3:

$$c_1 H_{11} + c_2 H_{12} + c_3 H_{13} + c_4 H_{14} = E_E\, c_1 \qquad (4)$$

Repeating the same procedure starting from Supplementary Equation 2 and multiplying it with $\varphi_1 \varphi^*_2 \varphi_3 \varphi_4$, $\varphi_1 \varphi_2 \varphi^*_3 \varphi_4$ and $\varphi_1 \varphi_2 \varphi_3 \varphi^*_4$, respectively, one obtains the following equations:

$$c_1 H_{21} + c_2 H_{22} + c_3 H_{23} + c_4 H_{24} = E_E c_2 \quad (5)$$

$$c_1 H_{31} + c_2 H_{32} + c_3 H_{33} + c_4 H_{34} = E_E c_3 \quad (6)$$

$$c_1 H_{41} + c_2 H_{42} + c_3 H_{43} + c_4 H_{44} = E_E c_4 \quad (7)$$

Supplementary Equations 4–7 can be summarized in a matrix notation, leading to Supplementary Equation 8 which represents an eigenwert equation.

$$\begin{pmatrix} H_{11} & H_{12} & H_{13} & H_{14} \\ H_{21} & H_{22} & H_{23} & H_{24} \\ H_{31} & H_{32} & H_{33} & H_{34} \\ H_{41} & H_{42} & H_{43} & H_{44} \end{pmatrix} \begin{pmatrix} c_1 \\ c_2 \\ c_3 \\ c_4 \end{pmatrix} = E_E \begin{pmatrix} c_1 \\ c_2 \\ c_3 \\ c_4 \end{pmatrix} \quad (8)$$

Using the next-neighbor approximation certain matrix elements can be set to zero since only the interaction between chromophores next to each other are taken into account.[1] Thus, the Hamiltonian matrix simplifies to:

$$\begin{pmatrix} H_{11} & H_{12} & 0 & 0 \\ H_{21} & H_{22} & H_{23} & 0 \\ 0 & H_{32} & H_{33} & H_{34} \\ 0 & 0 & H_{43} & H_{44} \end{pmatrix}$$

In the following, the non-diagonal matrix elements are denoted by J_{xy} (with $J_{xy} = J_{yx}$) representing the exciton coupling energies. Furthermore, the diagonal elements H_{11}, H_{22}, H_{33} and H_{44} represent the excited state energies of the corresponding chromophores. Thus, the Hamiltonian operator for the quadruple chromophore stack of bis(merocyanine) **1** has the following form

$$\begin{pmatrix} 0 & J_{12} & 0 & 0 \\ J_{12} & 0 & J_{23} & 0 \\ 0 & J_{23} & 0 & J_{34} \\ 0 & 0 & J_{34} & 0 \end{pmatrix}$$

where the excited state energy of the monomeric short chromophore is set as zero level. Due to the centrosymmetry of the dye stack the matrix simplifies to

$$\begin{pmatrix} 0 & J_{12} & 0 & 0 \\ J_{12} & 0 & J_{23} & 0 \\ 0 & J_{23} & 0 & J_{12} \\ 0 & 0 & J_{12} & 0 \end{pmatrix}$$

since the exciton coupling energies J_{12} and J_{34} are equal.

In an analogous manner the Hamiltonian matrix for the dimer of bis(merocyanine) **2** can be written as

$$\begin{pmatrix} \Delta E & J_{12} & 0 & 0 \\ J_{12} & -\Delta E & J_{23} & 0 \\ 0 & J_{23} & -\Delta E & J_{12} \\ 0 & 0 & J_{12} & \Delta E \end{pmatrix}$$

where ΔE is half the energy difference between the first excited states of the monomeric long and short chromophore, respectively. In addition, the midpoint of the excited state energies of both chromophores is set as zero level.

The excited state energies of the quadruple dye stacks correspond to the eigenvalues of the Hamiltonian matrices. For the dye stack of bis(merocyanine) **1** we obtain:

$$\begin{aligned} E_{\mathrm{E1}} &= \frac{1}{2}(-\sqrt{4J_{12}^2 + J_{23}^2} - J_{23}) \\ E_{\mathrm{E2}} &= \frac{1}{2}(-\sqrt{4J_{12}^2 + J_{23}^2} + J_{23}) \\ E_{\mathrm{E3}} &= \frac{1}{2}(\sqrt{4J_{12}^2 + J_{23}^2} - J_{23}) \\ E_{\mathrm{E4}} &= \frac{1}{2}(\sqrt{4J_{12}^2 + J_{23}^2} + J_{23}) \end{aligned}$$

and for bis(merocyanine) **2**:

$$\begin{aligned} E_{\mathrm{E1}} &= \frac{1}{2}(-J_{23} - K) \\ E_{\mathrm{E2}} &= \frac{1}{2}(J_{23} - L) \\ E_{\mathrm{E3}} &= \frac{1}{2}(-J_{23} + K) \\ E_{\mathrm{E4}} &= \frac{1}{2}(J_{23} + L) \end{aligned}$$

with $L = \sqrt{4J_{12}^2 + 4\Delta E^2 - 4\Delta E J_{23} + J_{23}^2}$

and $K = \sqrt{4J_{12}^2 + 4\Delta E^2 + 4\Delta E J_{23} + J_{23}^2}$

Thus, in order to calculate the excited state energies one has to determine the exciton coupling energies of two neighbored chromophores in the stack as well as the excited state energies of the isolated chromophores.

The latter can be obtained from the corresponding absorption maxima of the ideal monomer spectrum of the corresponding bis(merocyanine) dyes **1** and **2** obtained by concentration-dependent UV/Vis spectroscopy in chlorobenzene. In this way, we obtain an excited state energy of 18505 cm^{-1} for the chromophore of bis(merocyanine) **1** and 18229 cm^{-1} for the short chromophore and 15084 cm^{-1} for the long chromophore of bis(merocyanine) **2**, respectively.

The exciton coupling energy between two next-neighbored chromophores of the quadruple dye stacks was determined by applying the transition charge method.[2,3] For this, a Mulliken population analysis of the transition densities of the chromophores obtained by TDDFT calculations (ωB97/def2-SVP) was performed using the Multiwfn software package[4] to derive the transition charges (Supplementary Table 4 and 5). The magnitudes of the transition dipole moments calculated from the transition charges (9.7 D for the short chromophore and 12.2 D for the long chromophore, respectively) are in very good agreement with the magnitudes of the transition dipole moment obtained directly by TDDFT calculations (Supplementary Fig. 19c) as well as with experimental values (9.8 D for the short chromophore and 12.3 D for the long chromophore, respectively)[5]. In addition, the orientations of the transition dipole moments calculated from transition charges match with the orientations of the transition dipole moments calculated directly by TDDFT calculations. Thus, the transition charges obtained by Mulliken population analysis of the transition densities are suitable to calculate the exciton coupling energies between the merocyanine chromophores which are defined as the Coulomb interaction between the transition charges of the respective chromophores. Using the geometry optimized structures of both dimer aggregates we obtain the results listed

in Supplementary Table 6. With this, one can calculate the excited state energies of the dye stacks of bis(merocyanine) dyes **1** and **2** (Supplementary Table 7).

The overall transition dipole moment ($\boldsymbol{\mu}_{eg}$) of the aggregate structure can be written as a linear combination of the transition dipole moments of the individual chromophores:

$$\boldsymbol{\mu}_{eg} = c_1 \cdot \boldsymbol{\mu}_{eg,1} + c_2 \cdot \boldsymbol{\mu}_{eg,2} + c_3 \cdot \boldsymbol{\mu}_{eg,3} + c_4 \cdot \boldsymbol{\mu}_{eg,4} \quad (9)$$

where the coefficients c_1–c_4 are the same as shown in Supplementary Equation 1, and thus can be determined by calculating the normed eigenvectors of the corresponding Hamiltonian matrix (Supplementary Note 1 and 2). The calculated values are listed in Supplementary Table 8 and 9.

Since the ground state dipole moments of the chromophores are aligned antiparallely within the stacks, one can assume that all the transition dipole moments of the chromophores oscillate with the same orientation because the ground state dipole moment and the transition dipole moment of a merocyanine chromophore are aligned along the same axis (Supplementary Fig. 19c). Consequently, the magnitude of the overall transition dipole moment ($|\boldsymbol{\mu}_{eg}|$) of the aggregate structure can be calculated from Supplementary Equation 9 by using the magnitudes of the transition dipole moments of the individual chromophores.

Supplementary Methods

Synthesis and characterization

Synthesis of 3-ethylthiophene-2-carboxaldehyde (8)[6]

A mixture of dimethylformamide (520 µL, ρ = 0.948 g/mL, 493 mg, 6.74 mmol) and phosphorus oxychloride (624 µL, ρ = 1.65 g/mL, 1.03 g, 6.73 mmol) was stirred until the heat evolution was ceased. Afterwards, 3-ethylthiophene (500 mg, 4.46 mmol) was added dropwise over a period of 1 h. The mixture was stirred for 1 h at room temperature and for additional 1 h at 100 °C. After cooling down to room temperature, dichloromethane (50 mL) and a saturated aqueous solution of $NaHCO_3$ (50 mL) were added and the layers were separated. The aqueous layer was extracted with dichloromethane (3 x 30 mL) and the organic layers were combined and washed with brine (50 mL). The solvent was removed under reduced pressure and the crude product was purified by column chromatography (silica gel, hexane/ethyl acetate 9:1) to afford a bright yellow oil (724 mg, 3.88 mmol, 87%) consisting of a 3:1 mixture of the regioisomers 3-ethylthiophene-2-carboxaldehyde **8** and 4-ethylthiophene-2-carboxaldehyde **9**. The regioisomeric ratio was determined by ^{1}H NMR spectroscopy of the mixture (Supplementary Fig. 1). ^{1}H NMR of **8** (400 MHz, $CDCl_3$): δ 10.0 (d, ^5J = 1.1 Hz, 1H), 7.64 (dd, ^3J = 5.0 Hz, ^5J = 1.1 Hz, 1H), 7.03 (d, ^3J = 5.0 Hz, 1H), 3.00 (q, ^3J = 7.6 Hz, 2H), 1.31 (t, J = 7.6 Hz, 3H). ^{1}H NMR of **9**: ^{1}H NMR (400 MHz, $CDCl_3$): δ 9.87 (d, ^5J = 1.2 Hz, 1H), 7.62 (m, 1H), 7.38 (m, 1H), 2.68 (m, 2H), 1.27 (t, ^3J = 7.5 Hz, 3H).

Synthesis of 5-bromo-3-ethylthiophene-2-carboxaldehyde (10)

A portion of 400 mg (2.86 mmol) of a 3:1 mixture of 3-ethylthiophene-2-carboxaldehyde **8** and 4-ethylthiophene-2-carboxaldehyde **9**, as obtained from the previous reaction, was dissolved in chloroform (15 mL) and cooled to 0 °C. Bromine (150 µL, ρ = 3.10 g/mL, 465 mg, 2.91 mmol) was added dropwise over a period of 15 min and the mixture stirred for

2 h at room temperature. A saturated aqueous solution of $Na_2S_2O_3$ (5 mL) and dichloromethane (100 mL) were added and the layers were separated. The organic layer was washed successively with distilled water (50 mL), aqueous solution of K_2CO_3 (50 mL), and brine (50 mL). The solvent was removed under reduced pressure and the crude product was purified by column chromatography (silica gel, hexane/ethyl acetate 19:1) to afford a bright yellow oil (519 mg, 2.37 mmol, 83%) consisting of a 3:1 mixture of the regioisomers 5-bromo-3-ethylthiophene-2-carboxaldehyde **10** and 5-bromo-4-ethylthiophene-2-carboxaldehyde **11**. The regioisomeric ratio was determined by ^{1}H NMR spectroscopy of the mixture (Supplementary Fig. 2). ^{1}H NMR of **10** (400 MHz, $CDCl_3$): δ 9.91 (s, 1H), 7.02 (s, 1H), 2.95 (q, $^3J = 7.6$ Hz, 2H), 1.30 (t, $^3J = 7.6$ Hz, 3H). ^{1}H NMR of **11** (400 MHz, $CDCl_3$): δ 9.76 (s, 1H), 7.48 (s, 1H), 2.63 (q, $^3J = 7.6$ Hz, 2H), 1.24 (t, $^3J = 7.6$ Hz, 3H). Anal. calcd. for C_7H_7BrOS: C, 38.37; H, 3.22; S, 14.63. Found: C, 38.46; H, 3.28; S, 14.76 (for regioisomeric mixture). HRMS (ESI): *m/z* 218.94743 $[M\text{+H}]^+$, calcd. for $C_7H_8BrOS^+$: 218.94737 (for regioisomeric mixture).

Synthesis of 5-(dibutylamino)-3-ethylthiophene-2-carboxaldehyde (5)

To a portion of 490 mg (2.24 mmol) of a 3:1 mixture of 5-bromo-3-ethylthiophene-2-carboxaldehyde **10** and 5-bromo-4-ethylthiophene-2-carboxaldehyde **11**, as obtained from the previous reaction, and dibutylamine (700 mg, 5.43 mmol) was added dimethylformamide (3 mL) and distilled water (1 mL). The mixture was then stirred for 2 h at 130 °C in the microwave. Distilled water (100 mL) and dichloromethane (100 mL) were added and the organic phase was separated. The aqueous layer was extracted with dichloromethane (2 x 70 mL) and the organic phases were combined. The solvent was removed under reduced pressure and the crude product was purified by column chromatography (silica gel, dichloromethane/ethyl acetate 8:1) yielding compound **5** as the only product as a brown oil

(204 mg, 45% referring to corresponding major regioisomer **10**). ^{1}H NMR (400 MHz, CD_2Cl_2): δ 9.60 (s, 1H), 5.77 (s, 1H), 3.31 (t, ^3J = 7.7 Hz, 4H), 2.80 (q, ^3J = 7.5 Hz, 2H), 1.63 (m, 4H), 1.36 (m, 4H), 1.26 (t, ^3J = 7.5 Hz, 3H), 0.96 (t, ^3J = 7.4 Hz, 6H). Anal. calcd. for $C_{15}H_{25}NOS$: C, 67.37; H, 9.42; N, 5.24; S, 11.99. Found: C, 67.63; H, 9.50; N, 5.30; S, 11.79. HRMS (ESI): *m/z* 268.17272 [*M*+H]$^+$, calcd. for $C_{15}H_{26}NOS$: 268.17296.

Synthesis of bis(merocyanine) 1

Bis(pyridone) **3** [7] (150 mg, 251 μmol) and 5-(dibutylamino)thiophene-2-carbaldehyde **4**[5] (132 mg, 551 μmol) were stirred for 1 h in acetic anhydride (5 mL) at 90 °C. The mixture was cooled down to room temperature, methanol (10 mL) and ethanol (10 mL) were added to the reaction mixture and the resulting solution was concentrated in vacuo. The crude product was purified by column chromatography (silica gel, CH_2Cl_2/MeOH 98:2) to give bis(merocyanine) dye **1** (143 mg, 138 μmol, 55%) as a red solid. Mp: 196–198 °C; ^{1}H NMR (600 MHz, CD_2Cl_2, 295 K): δ 7.41 (m, 4H), 7.12 (s, 2H), 6.92 (s, 2H), 6.36 (d, ^3J = 5.2 Hz, 2H), 5.17 (s, 4H), 4.17 (t, ^3J = 6.5 Hz, 4H), 3.46 (t, ^3J = 7.7 Hz, 8H), 2.28 (br. s, 6H), 1.94–1.89 (m, 4H), 1.68–1.60 (m, 12H), 1.37–1.34 (m, 8H), 1.04 (t, ^3J = 7.4 Hz, 6H), 0.92 (t, ^3J = 7.3 Hz, 12H); ^{13}C NMR (151 MHz, CD_2Cl_2): δ 177.1, 163.6, 162.3, 159.0, 155.6, 152.4, 141.4, 134.2, 125.1, 124.8, 123.1, 122.6, 117.6, 112.4, 106.5, 105.2, 94.1, 68.2, 39.3, 31.8, 31.0, 29.6, 20.5, 19.9, 19.2, 14.2, 13.9; UV/Vis (CH_2Cl_2): λ_{max} (ε) = 538 nm (278000 M^{-1} cm^{-1}). HRMS (ESI): *m/z* 1039.5177 [*M*+H]$^+$, calcd. for $C_{60}H_{75}N_6O_6S_2^+$: 1039.5184.

Synthesis of bis(merocyanine) 2

Bis(pyridone) **3**[7] (78.0 mg, 131 µmol), 5-(dibutylamino)-3-ethylthiophene-2-carboxaldehyde **5** (42.0 mg, 157 µmol) and aminothiophene **6**[5] (41.0 mg, 155 µmol) were stirred for 2 h in acetic anhydride (1 mL) at 100 °C. The solvent was removed under reduced pressure and the crude product was purified by column chromatography (silica gel, dichloromethane/ethyl acetate 8:1) yielding a purple solid (14.3 mg, 13.1 µmol 10%). Mp: 182-184 °C; ^{1}H NMR (400 MHz, CD_2Cl_2): δ 7.99 (br, 1H), 7.70 (s, 1H), 7.38-7.30 (m, 3H), 7.07 (s, 2H), 6.93 (s, 1H), 6.91 (s, 1H), 6.28 (s, 1H), 6.20 (d, ^3J = 4.4 Hz, 1H), 5.17 (s, 4H), 4.15 (t, ^3J = 6.3 Hz, 4H), 3.43 (m, 8H), 2.80 (q, ^3J = 7.6 Hz, 2H), 2.49 (s, 3H), 2.37 (s, 3H), 1.93-1.87 (m, 4H), 1.70-1.58 (m, 11H), 1.37-1.27 (m, 12H), 1.04 (t, ^3J = 7.3 Hz, 3H), 1.03 (t, ^3J = 7.3 Hz, 3H), 0.95-0.96 (m, 12H); ^{13}C NMR (151 MHz, CD_2Cl_2): δ 176.8, 175.1, 171.9, 167.9, 163.6, 163.4, 162.7, 162.6, 158.7, 158.2, 155.6, 155.5, 151.6, 148.9, 145.0, 136.8. 134.1, 129.4, 128.5, 127.8, 125.6, 124.9, 124.8, 123.3, 123.0, 121.3, 119.0, 118.2, 117.9, 112.7, 111.5, 110.9, 105.8, 105.0, 94.6, 93.2, 68.07, 68.06, 55.1, 39.4, 39.2, 31.82, 31.81, 29.5, 24.0, 20.5, 20.4, 19.91, 19.89, 19.2, 19.0, 14.8, 14.18, 14.16, 13.9; UV/Vis (CH_2Cl_2): λ_{max} (ε) = 541 nm (126000 M^{-1} cm^{-1}), 678 nm (135000 M^{-1} cm^{-1}). HRMS (ESI): *m/z* 1093.56763 [*M*+H]$^+$, calcd. for $C_{64}H_{81}N_6O_6S_2^+$: 1093.56535.

Supplementary References

1. Lambert, C., Scherpf, T., Ceymann, H., Schmiedel, A. & Holzapfel, M. Coupled oscillators for tuning fluorescence properties of squaraine dyes. *J. Am. Chem. Soc.* **137**, 3547–3557 (2015).
2. Chang, J. C. Monopole effects on electronic excitation interactions between large molecules. I. Application to energy transfer in chlorophylls. *J. Chem. Phys.* **67**, 3901–3909 (1977).
3. Kistler, K. A., Spano, F. C. & Matsika, S. A benchmark of excitonic couplings derived from atomic transition charges. *J. Phys. Chem. B* **117**, 2032–2044 (2013).
4. Lu, T. & Chen, F. Multiwfn: A multifunctional wavefunction analyzer. *J. Comp. Chem.* **33**, 580–592 (2012).
5. Zitzler-Kunkel, A. *et al.* NIR-absorbing merocyanine dyes for BHJ solar cells. *Chem. Mater.* **26**, 4856–4866 (2014).
6. Detty, M. R. & Hays, D. S. Studies towards alkylthiophene-2-carboxaldehydes. Reduction of 3-alkenylthiophenes with triethylsilane/trifluoroacetic acid. Regioselectivity in formylation reactions of 3-alkylthiophenes. *Heterocycles* **40**, 925–937 (1995).
7. Lohr, A., Grüne, M. & Würthner, F. Self-Assembly of bis(merocyanine) tweezers into discrete bimolecular π-stacks. *Chem. Eur. J.* **15**, 3691–3705 (2009).
